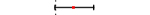# Simultaneous multifactor Bayesian analysis (SiMBA) of PET time activity curve data

**DOI:** 10.1016/j.neuroimage.2022.119195

**Published:** 2022-04-19

**Authors:** Granville J. Matheson, R. Todd Ogden

**Affiliations:** a Department of Psychiatry, Columbia University, New York, NY 10032, USA; b Department of Biostatistics, Columbia University Mailman School of Public Health, New York, NY 10032, USA

## Abstract

Positron emission tomography (PET) is an *in vivo* imaging method essential for studying the neurochemical pathophysiology of psychiatric and neurological disease. However, its high cost and exposure of participants to radiation make it unfeasible to employ large sample sizes. The major shortcoming of PET imaging is therefore its lack of power for studying clinically-relevant research questions. Here, we introduce a new method for performing PET quantification and analysis called SiMBA, which helps to alleviate these issues by improving the efficiency of PET analysis by exploiting similarities between both individuals and regions within individuals. In simulated [^11^C]WAY100635 data, SiMBA greatly improves both statistical power and the consistency of effect size estimation without affecting the false positive rate. This approach makes use of hierarchical, multifactor, multivariate Bayesian modelling to effectively borrow strength across the whole dataset to improve stability and robustness to measurement error. In so doing, parameter identifiability and estimation are improved, without sacrificing model interpretability. This comes at the cost of increased computational overhead, however this is practically negligible relative to the time taken to collect PET data. This method has the potential to make it possible to test clinically-relevant hypotheses which could never be studied before given the practical constraints. Furthermore, because this method does not require any additional information over and above that required for traditional analysis, it makes it possible to re-examine data which has already previously been collected at great expense. In the absence of dramatic advancements in PET image data quality, radiotracer development, or data sharing, PET imaging has been fundamentally limited in the scope of research hypotheses which could be studied. This method, especially combined with the recent steps taken by the PET imaging community to embrace data sharing, will make it possible to greatly improve the research possibilities and clinical relevance of PET neuroimaging.

## 1. Introduction

PET quantification involves fitting pharmacokinetic (PK) models to a series of radioactivity concentrations in a region of the brain over time, called a time activity curve (TAC). Fitting these models provide estimates of binding, which are generally assumed to be proportional to the density of the target molecule. Typically, these models are first fitted separately to each TAC from each brain region of each individual to derive estimates of binding, and these binding estimates are subsequently compared between individuals. While statistically valid, this approach represents an inefficient use of the acquired data due to the fact that no information can be retained between TACs: the model effectively forgets everything it has learnt when presented with each new TAC from each new individual (McElreath, 2016; 2017). Allowing a model to make use of more data at once is a natural strategy by which to improve the ability of PET imaging to infer upon difficult-to-estimate-parameters. For instance, Simultaneous Estimation of *V*_ND_, SIME-*V*_ND_ (Ogden et al., 2015) is a method designed to estimate the degree of nondisplaceable binding at the individual level, which is otherwise estimated poorly, by fitting data from multiple regions at once and assuming a common value of this parameter.

The conventional strategy of fitting a unique set of parameters to each TAC *independently* of all the others, is therefore referred to as a “no pooling” approach. The opposite extreme is that of “complete pooling,” in which a common parameter, or set of parameters, are fitted to all TACs simultaneously, which considers all TACs as effectively *interchangeable* for the estimation of completely pooled parameters. SIME-*V*_ND_ (Ogden et al., 2015), for example, makes use of complete pooling between regions for the estimation of nondisplaceable binding, and no pooling for the estimation of the remaining parameters. However, in most circumstances, it is neither appropriate to assume complete independence nor complete interchangeability of pharmacokinetic parameters between regions or individuals.

“Partial pooling” serves to make a compromise between these two approaches, by considering individual parameters to be drawn from a common overarching population distribution. By estimating both population and individual parameters simultaneously, the model is able to flexibly determine the degree of pooling consistent with the observed data. This results in adaptively regularised estimates which are shrunk towards the global mean, thereby achieving a balance between the above two strategies; considering the data as neither completely independent nor completely interchangeable. This allows the model to exploit similarities between data from different sources, i.e., from different regions and individuals, within the sample, and thereby “borrow strength,” leading to improved estimates and predictions, as well as increased power for hypothesis testing (Gelman and Hill, 2007). Hierarchical or mixed effects modelling, which make use of partial pooling, is routinely applied in numerous and diverse fields of research, and is often regarded as a more sensible default method by which to perform statistical inference in general whenever a common overarching distribution can be assumed (McElreath, 2017).

In addition to improved estimation, a further advantage of these types of models is that estimation and statistical analysis can be performed simultaneously. The conventional strategy can be described as a two stage approach: first, parameters are estimated from each individual TAC, and subsequently statistical analysis is performed using the point estimate of the relevant estimated parameter, e.g. comparing estimates of specific binding between groups of patients and controls. Combining estimation and statistical analysis within a hierarchical model has several advantages. Firstly, this allows the uncertainty in estimated parameters to be propagated to the statistical analysis, resulting in improved power. Secondly, this allows for the covariance structure between the estimated pharmacokinetic parameters to be utilised to stabilise one another during the statistical analysis, i.e. if two pharmacokinetic parameters are highly correlated with one another, then the estimate for the first parameter provides the model with relevant information with which to guide the estimation of the second parameter. Taken together, in contrast to the two-stage approach, a hierarchical modelling strategy is able to gain additional statistical power by performing parameter estimation and statistical modelling simultaneously, over and above the increases in power gained from improved parameter estimation owing to partial pooling.

Almost all PET PK models used in practice are simplifications of more complex, but more biologically appropriate models. While more complex models likely reflect the underlying biology more accurately, their complexity, i.e. the number of parameters fitted, impedes their stability and accuracy. For this reason, almost all kinetic modelling innovations impose additional assumptions to reduce the number of free parameters estimated, and thereby fit a simpler model to estimate the same thing as the more complex model, but with greater robustness. For instance, the simplified reference tissue model (Lammertsma and Hume, 1996; Salinas et al., 2014) assumes that one-tissue compartment model dynamics hold in both the target and reference regions in order to eliminate one of the parameters of the full reference tissue model (Cunningham et al., 1991). These assumptions are rarely, if ever, strictly true; however they allow the model to better compensate for measurement noise and the limited information available in any single TAC to provide more stable estimates of binding. In other words, we tolerate the hopefully negligible bias induced by these assumptions in order to reduce the variance of our estimates by reducing the complexity of our models. Model complexity can be characterised as an overfitting risk, and is described by the penalty term in the calculation of information criteria: in the Akaike Information Criterion for example, this penalty is proportional to the number of free parameters in the model (Gelman et al., 2014). However, in the context of hierarchical modelling or informative priors in a Bayesian setting, the imposed regularisation lowers the risk of overfitting, and improves the identifiability and stability of parameter estimation. Hence, despite, in the case of hierarchical modelling, an increased number of estimated parameters in absolute terms due to estimation of both population and individual parameters; the number of *effective* parameters can be reduced considerably due to the adaptive regularisation imposed by the partial pooling. This therefore has the same effect as model simplification, but without making compromises at the level of the pharmacokinetic model itself.

A new preliminary application of partial pooling for PET PK modelling, with simultaneous parameter estimation and statistical comparison, was recently developed and evaluated (Chen et al., 2019). This approach demonstrated marked improvements over the conventional approach in both simulated and real data. However, its implementation using existing nonlinear mixed effects tooling (Pinheiro et al., 2021) has limited flexibility, thereby limiting the scope and complexity of the data-generating process which can be modelled using this approach. This means that it is only possible to apply this approach across either individuals in one region, or across regions within one individual. However, it is known that there is a great deal of additional information available from different regions of the brain within each individual, which is exploited by several different PK models (Ogden et al., 2015; Schain et al., 2017; 2018; Slifstein et al., 2015).

In this study, we present a novel hierarchical PK modelling framework, called SiMBA: Simultaneous Multifactor Bayesian Analysis. This framework allows for the application of the two-tissue compartment using a hierarchical multifactor model, meaning that partial pooling is applied across multiple, overlapping hierarchies of individuals, regions, and regions within individuals, while simultaneously performing statistical inference of pharmacokinetic parameters. We make use of Bayesian techniques in order to allow us to make use of complex variance-covariance structures, as well as to be able to incorporate prior information. We demonstrate our results in both simulated and real data.

## 2. Methods

### 2.1. Pharmacokinetic model

The TAC measured by the PET system consists of a series of measurements of the concentration of radioactivity over time (*t*). In PET modelling, the TAC is conceptualised as the total (T) radioactivity concentration (C) within the region of interest, i.e. C_T_(t). This also includes a fractional blood volume contribution (*ν*_B_) from the small proportion of the volume comprised of blood. Hence, the model includes measurements of the radioactivity concentration in whole blood (C_B_(t)) which are measured at same time as the PET examination from drawn blood. The concentration in the tissue is described by the convolution of an arterial input function (AIF) and an impulse response function (IRF). The AIF, like the TAC, consists of a series of measurements over time of the concentration of radioactivity in the arterial plasma (C_P_(t)), after correction to account for the proportion of this radioactivity which is attributable to unmetabolized parent compound. The AIF therefore represents the concentration of the tracer which is available to enter the brain at each time point (although this does not account for plasma binding). The general PET pharmacokinetic modelling framework is therefore described as follows:

(1) $$C_{\text{T}}(t)=\left(1-v_{B}\right)\left(IRF⊗C_{\text{P}}\right)(t)+v_{\text{B}}C_{\text{B}}(t)=TCM(\theta ,\;t)$$

The whole tissue compartment model is abbreviated TCM, with the parameters contained within the vector *θ*.

**Fig. 1.**
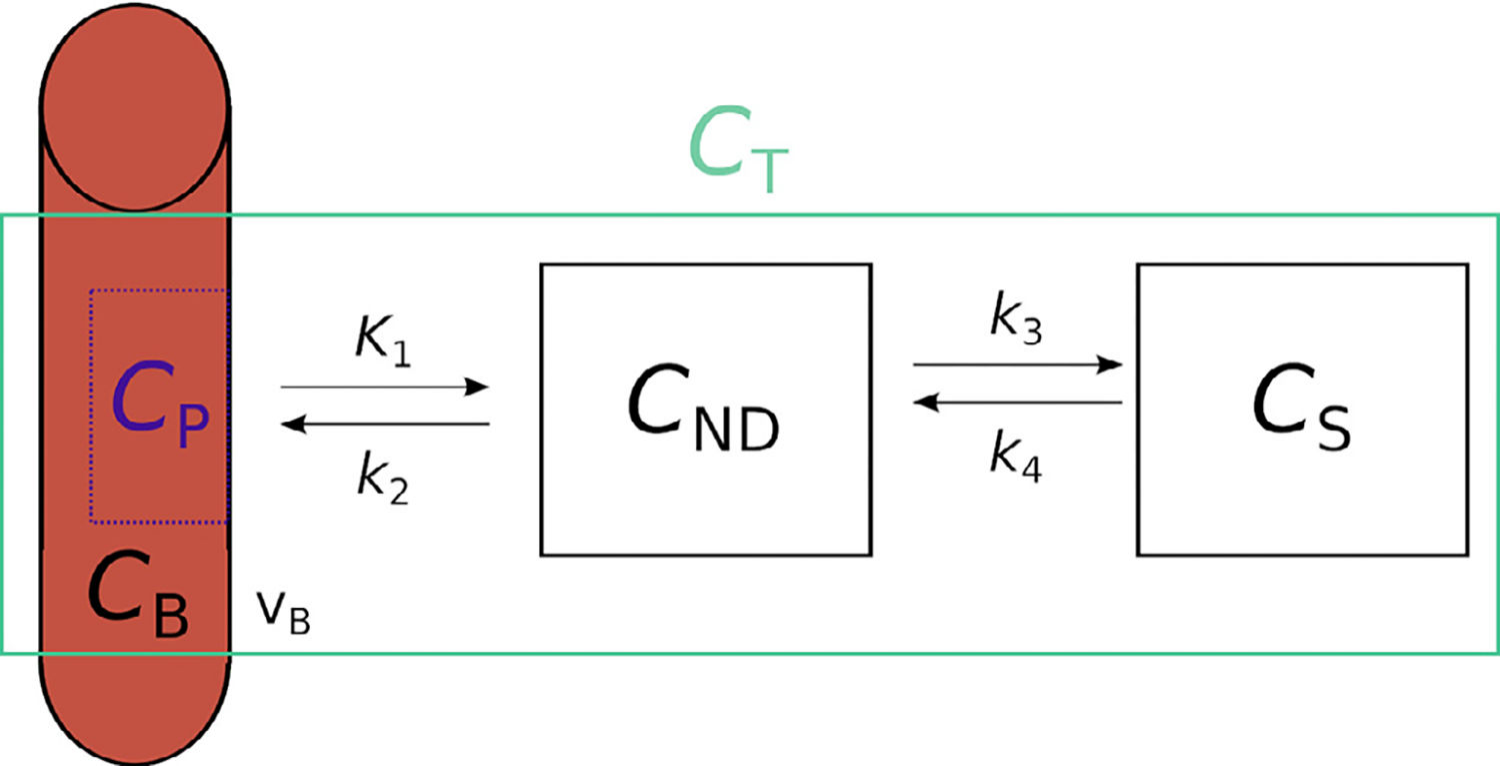
Two tissue compartment model schematic diagram. K_1_, k_2_, k_3_ and k_4_ represent the rate constants representing the rate of transfer between compartments. C represents the concentration of radioactivity in each compartment, in the specific compartment (S), non-displaceable compartment (ND), in the whole blood (B), and in the metabolite-corrected arterial plasma (P). The total (T) radioactivity concentration recorded using the PET system is represented with the green box. Blood vasculature is represented by the cross-section of the red artery, representing the blood volume fraction (v_B_) measured by the PET system, and included in C_T_.

The AIF, the whole blood radioactivity, and the TAC are measured, while the IRF may only be estimated from these other quantities, providing a description of the behaviour of the compound in the tissue as a function of its binding. In this study, we focus on the two-tissue compartment (2TC) model with five free parameters: rate constants *K*_1_, *k*_2_, *k*_3_, *k*_4_ and blood volume fraction *ν*_B_ (Fig. 1) Innis et al. (2007).

In this model the compartments represent the non-displaceable (ND) compartment, itself comprised of non-specifically bound and free compartments, and the specific (S) compartment. Their volumes of distribution (V) refer to the concentration of a given compartment (represented by the subscript) at equilibrium relative to the metabolite-corrected arterial plasma (P). Alternatively, binding potential (BP) refers to the specific binding defined relative to the concentration of other compartments, represented by subscripts, at equilibrium. These quantities of biological interest can also be expressed as functions of the rate constants:

(2) $$V_{T}=\frac{K_{1}}{k_{2}}\left(1+\frac{k_{3}}{k_{4}}\right)\;V_{ND}=\frac{K_{1}}{k_{2}}\;BP_{P}=V_{S}=\frac{K_{1}k_{3}}{k_{2}k_{4}}\;BP_{ND}=\frac{k_{3}}{k_{4}}$$

### 2.2. Generalised model framework

Traditionally, the pharmacokinetic model would be fitted to each TAC individually using weighted nonlinear least squares (NLS). The weights are usually calculated such that they are approximately proportional to the inverse variance of the measurement error in each frame, and there exist several different proposed methods for calculating weights (Thiele and Buchert, 2008). The estimated set of parameters thus represent the maximum likelihood estimate, i.e., the set of parameter values which maximise the likelihood of the data, *P* (*data*|*θ*), which, when a Gaussian distribution is assumed for the observations, minimises the sum of squared weighted residuals. We can hence describe the theoretical data generating process for the model described in Eq. 1, here considering a single region of interest (ROI) from a single subject, for each time frame *i*, as follows:

$${\text{C}}_{T}\left(t_{\left[i\right]}\right)\sim \text{Normal}\left(\mu_{\left[i\right]},\;\sigma_{\left[i\right]}^{2}\right)$$

$$\mu_{\left[i\right]}=\mathrm{TCM}\left(\theta ,\;t_{\left[i\right]}\right)$$

$$\sigma_{\left[i\right]}=\frac{1}{\sqrt{w_{\left[i\right]}}}\sigma$$

where *μ* represents the estimated TAC value, and *σ* the standard deviation of the error of the TAC, where *σ*_[*i*]_ refers to the error for the each frame of the PET measurement. The model weights, *w*, are calculated *a priori*.

Indices are presented in square brackets throughout to avoid ambiguity in later equations where pharmacokinetic parameters are also specified as subscripts.

**Fig. 2.**
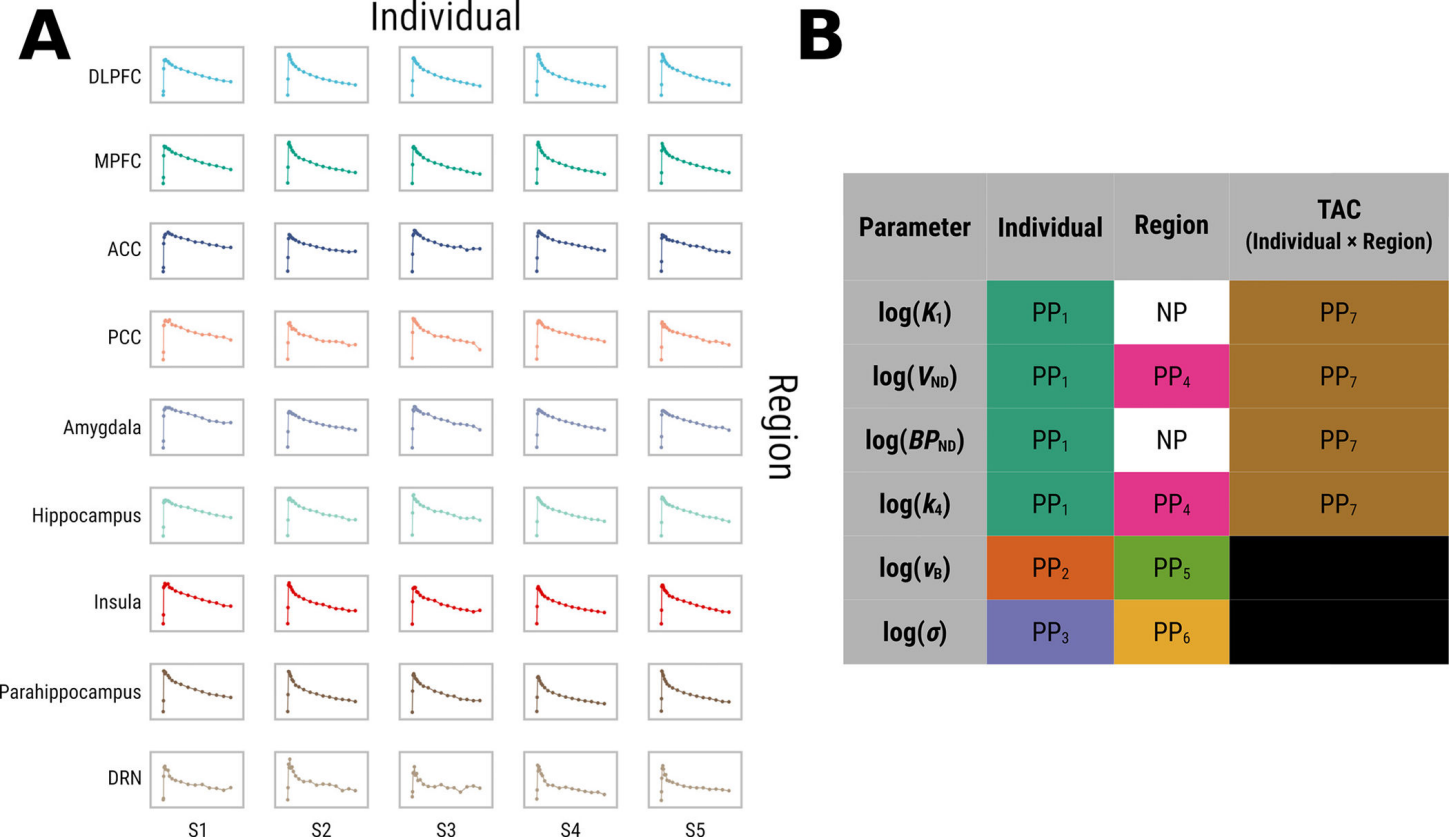
The structure of the data and of the model. Panel A: TACs are available for each region of each individual. The parameters of the model at the level of individuals are estimated in common for all regions (i.e. for columns across rows), while region parameters are estimated in common for all individuals (i.e. for rows across columns). Only for the Individuals × Regions hierarchy are parameters are estimated for each TAC within each grey box. Panel B: Parameters are estimated using either partial pooling (PP) or no pooling (NP). White squares represent no pooling, while coloured squares represent partial pooling, coloured by the particular one of the seven variance-covariance estimated matrices the parameter belongs to, i.e. parameters for which the other parameters with the same colour can influence their values through their correlation matrix. Black squares indicate that no additional estimation was performed, or that the variance was set to 0, i.e. estimates were completely pooled using the estimates made at other levels. Regional abbreviations are as follows: DLPFC is dorsolateral prefrontal cortex, MPFC is medial prefrontal cortex, ACC is anterior cingulate cortex, PCC is posterior cingulate cortex, and DRN is dorsal raphe nucleus.

The model presented in Chen et al. (2019) is a hierarchical model in that it makes use of partial pooling across individuals by considering them to be drawn from a common overarching population distribution. In Fig. 2A, this model applies partial pooling across rows, but not columns.

For each time frame *i* and subject *j*, this model can be described as follows:

$${\text{C}}_{T}\left(t_{\left[i,j\right]}\right)\sim \text{Normal}\left(\mu_{\left[i,j\right]},\;\sigma_{\left[i,j\right]}^{2}\right)$$

$$\mu_{\left[i,j\right]}=\mathrm{TCM}\left(\theta_{j},\;t_{\left[i,j\right]}\right)$$

$$\theta_{\left[j\right]}\sim \mathrm{MVNormal}(\theta ,\;\Sigma )+X\beta$$

$$\Sigma =\left[\begin{array}{ccc} \sigma_{1} & 0 & 0\\ 0 & \ddots & 0\\ 0 & 0 & \sigma_{5} \end{array}\right]R\left[\begin{array}{ccc} \sigma_{1} & 0 & 0\\ 0 & \ddots & 0\\ 0 & 0 & \sigma_{5} \end{array}\right]$$

$$\sigma_{\left[i,j\right]}=\frac{1}{\sqrt{w_{\left[i\right]}}}\sigma$$

where Σ is the 5 × 5 covariance matrix for all of the pharmacokinetic parameters within the theta vector, which is decomposed into a correlation matrix **R** and a diagonal matrix of the standard deviations of each parameter. MVNormal refers to a multivariate normal distribution. Covariates, *X*, are represented by a n × p matrix multiplied by the 1 × p coefficient vector, *β*, where n represents the number of data points, and p represents the number of parameters. These covariates are unpooled parameters which are commonly referred to as fixed effects, in contrast to the partially pooled estimation of differences between individuals which are commonly referred to as random effects. Owing to substantial ambiguity between different fields in the use of these terms (Betancourt, 2020a), we will use the terms partially pooled and unpooled whenever possible to improve clarity.

Here, we extend this framework to accommodate not only TAC data from multiple subjects, but also multiple regions within each subject. To this end, we specify a multifactor model for each parameter within *θ*, defining a global mean intercept (*α*), a covariate vector (*β*) multiplied by a covariate matrix (*X*), and an additive sequence of residuals for each of the separate hierarchies (Betancourt, 2021): across individuals (*τ*_[*j*]_), across regions (*υ*_[*k*]_), and (*ϕ*_[*j,k*]_) for the interaction of regions and individuals, i.e. individual TACs.

$$\theta_{\left[j,k\right]}=\alpha_{\theta }+X\beta_{\theta }+\tau_{\theta [j]}+v_{\theta [k]}+\phi_{\theta [j,k]}$$

$$\tau \sim \text{MVNormal}\left([0],\;\Sigma_{\text{Subject}}\right)$$

$$v\sim \text{MVNormal}\left([0],\;\Sigma_{\text{Region}}\right)$$

$$\phi \sim \text{MVNormal}\left([0],\;\Sigma_{\text{TAC}}\right)$$

With respect to Fig. 2A, *τ* parameters are estimated in common for all rows across each column, *υ* parameters are estimated in common for all columns across each row, and finally *ϕ* parameters are estimated in common for each individual curve within the grey squares. In this way, the model defines a global average set of parameters for a hypothetical average individual and average region, together with individual deviations for a hypothetical average region, regional deviations for a hypothetical average individual, and finally any residual differences at the level of individual TAC curves, i.e. regions within individuals.

Similarly, the standard deviation of the measurement error, *σ* is log transformed and defined by a linear model, with a global mean intercept, covariate vector and matrix, and a sequence of terms for individual-specific, region-specific and TAC-specific differences. To account for differences in measurement error between the different frames within each TAC, a weighting function is also incorporated into the model. The optimal weights for weighted least squares estimation are proportional to the inverse variance of the measurement error (i.e.$w\propto \frac{1}{\sigma^{2}}$), so calculated weights values can be incorporated by first transforming them to measurement error (i.e.$\frac{1}{\sqrt{w}}$), taking their natural logarithm so that they can be a linear predictor for the log-transformed measurement error term, and centering them by subtracting the mean value within each individual. This weights-derived term is referred to below as w*. Region sizes and injected radioactivity can also be included in the covariate matrix.


$$\log\left(\sigma_{\left[i,j,k\right]}\right)=\alpha_{\sigma }+w_{\left[i,j,k\right]}^{*}+X\beta_{\sigma }+\tau_{\sigma [j]}+v_{\sigma [k]}+\phi_{\sigma [j,k]}$$

$$\tau_{\sigma }\sim \text{Normal}\left(0,\;\sigma_{\text{Individual}}^{2}\right)$$

$$v_{\sigma }\sim \text{Normal}\left(0,\;\sigma_{\text{Region}}^{2}\right)$$

$$\phi_{\sigma }\sim \text{Normal}\left(0,\;\sigma_{\text{TAC}}^{2}\right)$$

### 2.3. Model fitting

We make use of Bayesian hierarchical modelling to fit the model described above. In maximum likelihood estimation, a set of parameters are selected which maximise the likelihood of the data, i.e. *P*(*data*|*θ*). In contrast, Bayesian modelling assesses the probability of each set of parameters conditional on the data, i.e., *P*(*θ*|*data*). This allows full quantification of uncertainty of all parameters simultaneously, as well as for incorporating this uncertainty in the statistical analysis. Bayesian modelling is commonly performed using Markov Chain Monte Carlo (MCMC) sampling, which provides a great deal of flexibility for fitting large and complex models with complex covariance structures, which is required for fitting the complex multifactor model described above. This flexibility comes with some cost, however, as MCMC is highly computationally intensive.

## 3. Implementation

While the above section describes the general properties of such a model in theory, in this section we describe how it was implemented in practice.

### 3.1. Modelling of blood data

Arterial plasma radioactivity measurements and parent fraction measurements were collected as previously described (Parsey et al. (2005, 2010, 2000)). Parent fraction data were fitted using a Hill model, and a metabolite-corrected arterial plasma curve was created by taking the product of the estimated unmetabolised parent fraction and the arterial plasma radioactivity measurements. This curve was fitted using a linear rise followed by a sum of three exponentials to create the arterial input function (AIF).


$$AIF(t)=\{\begin{array}{ll} 0 & t<t_{0}\\ b\left(t-t_{0}\right) & t_{0}\leq t\leq t_{p}\\ \sum_{i=1}^{3}A_{i}e^{-\lambda_{i}\left(t-t_{0}\right)} & t>t_{p} \end{array}$$

The parameters include a delay term (t_0_, i.e., when the rise begins), a linear rise to the peak (gradient b, peaktime t_p_), followed by a sum of three exponential decay functions. The AIF was modelled using this function in order to make use of an analytical convolution with the IRF in the functional definition of the 2TC pharmacokinetic model for computational efficiency. Convolution using the Fast-Fourier Transform is not currently possible using the STAN modelling language (Carpenter et al., 2017), and numerical convolution is too inefficient to be considered a plausible alternative. In a future release, we hope to include the possibility of modelling the AIF using a spline function, analytically convolved with IRF for increased flexibility of SiMBA.

Whole blood radioactivity was not measured, so we made use of arterial plasma without parent fraction correction as a substitute for the whole blood radioactivity. Because the concentration of [^11^C]WAY100635 in red blood cells is negligible (Oikonen et al., 2000), this implies that the whole-blood-to-plasma ratio will remain constant throughout the measurement, and that the shape of the whole blood curve will not differ from that of the plasma. To avoid confusion with the metabolite-corrected arterial plasma, we will refer to this whole plasma curve as the whole blood curve. For kinetic modelling, we calculated average whole blood values for each PET time frame. For this, we fit the whole blood curve using the *kinfitr* spline blood model function (Matheson, 2019), which fits two splines: one for the rise to the peak, and another for the descent.

The fitted curve was then interpolated, and divided into segments corresponding to each frame of the PET examination after correcting for the delay between the curves. Mean whole blood concentrations during the course of each PET frame were calculated, which were used to represent the whole blood radioactivity for each frame during kinetic modelling.

The delay between the arterial input function and the TAC was fitted using the first 9 frames from the first six minutes of the PET measurement fit with a two-tissue compartment model (2TC) and an additional parameter for the delay using *kinfitr* (Matheson, 2019; Tjerkaski et al., 2020). Correspondence between the AIF and TAC were visually inspected, and when inadequate, the delay was selected using a semi-automatic approach in *kinfitr*, which identifies all local minima across a grid of potential delay values using a linearised 2TC model (Gjedde and Wong, 1990).

### 3.2. Pharmacokinetic model

For the purpose of facilitating the definition of priors, we parameterised the model to estimate *K*_1_, *V*_ND_, *BP*_ND_, *k*_4_ and *ν*_B_ using the relationships described in (2). *V*_ND_ and *BP*_ND_ were preferred over *k*_2_ and *k*_3_ since they have a more biologically interpretable meaning. This assists with the definition of priors in several ways. Firstly, *V*_ND_, in contrast to *K*_1_ or *k*_2_, is often assumed to be the same or similar between regions (Gunn et al., 2001; Ogden et al., 2015), or across individuals (Cunningham et al., 2009; Veronese et al., 2016). Secondly, *BP*_ND_ is theoretically proportional to the concentration of the target protein, assuming a same or similar *V*_ND_, and differences between groups can therefore be expressed in terms of a difference in *BP*_ND_. Additionally, this also served to allow for the setting of more conservative upper and lower limits in the traditional NLS analysis, thereby reducing the variability of outcome measures calculated from the pharmacokinetic rate constants.

Next, all parameters were transformed to their natural logarithms, serving two purposes. First, this naturally constrains all parameters to be positive, corresponding with their natural constraints as rate constants and biological quantities. Secondly, this serves to define additive differences within the model specification as proportional changes of the untransformed values. This is helpful because biological differences or changes in PET are typically assumed to exhibit similar proportional, but not absolute, differences between different regions, as the concentration of the protein of interest in different regions of the brain can differ by orders of magnitude. Indeed, multiplicative, as opposed to additive, relationships are more commonly observed in biology more generally (Gingerich, 2000; Xiao et al., 2011). Lastly, as a consequence of the log transformation, we are able to assume a common variance between regions, as the variance describes proportional differences, rather than absolute differences.

We made use of the two-tissue compartment model using an analytical convolution of the IRF with the parameters of the tri-exponential AIF, as well as the estimated scalar WB concentration values as described above.

### 3.3. Model specification

Section 2.2 laid out the most general framework for applying a 2TC kinetic model simultaneously across regions and across individuals. Next, we will illustrate the use of this model through one specific implementation and application to data. To adapt this general model to this particular situation, we describe here some specific choices we made.

As a general strategy, we estimated pharmacokinetic parameters from multivariate distributions in order to allow parameters to influence the estimation of one another through the correlation matrix. However, we opted to separate the blood volume fraction from the variance-covariance matrices of the other pharmacokinetic parameters because the former parameter is theoretically biologically independent of the other pharmacokinetic parameters, and should not be able to influence their estimation. Instead, the blood volume fraction and measurement error parameters were estimated using univariate partial pooling.

*K*_1_ and *BP*_ND_ are known to exhibit a substantial degree of heterogeneity between different regions as a result of well-understood biological differences, which can differ by orders of magnitude for some tracers and combinations of regions. For this reason, shrinking these *regional* differences towards a common mean (across regions) would not be appropriate. We therefore opted to estimate regional differences in *K*_1_ and *BP*_ND_ as unpooled parameters, i.e. fixed effects, by including regional differences as dummy variables within the covariate matrix. This estimates, for example, that the mean *BP*_ND_ in region B is 50% higher than for region A, but without considering a distribution of these differences. For *V*_ND_, *k*_4_ and *ν*_B_ on the other hand, there is no biological motivation, to our knowledge, to motivate extreme differences between regions. In fact, one model made use of complete pooling of both *V*_ND_ and *k*_4_ across regions (Slifstein et al., 2015), and *ν*_B_ is often estimated once per individual using a large region, or even set to 5% across an entire sample. Hence partial pooling of regional means was considered appropriate for these parameters.

Because the measurement error and blood volume fraction were not of primary interest, we made use of the expectation values estimated across regions and individuals, and did not estimate further residuals for TAC-specific differences. In other words, their *ϕ*_[*k*]_ terms were set to 0. For example, in Fig. 2, subject 3 appears to exhibit greater measurement error compared to the other individuals, hence *τ*_*σ*[*S*3]_ would be positive. Similarly the dorsal raphe nucleus (DRN) appears to exhibit greater measurement error than the other regions on average, and so *υ*_*σ*[*DRN*]_ would also be positive. The mean expectation value for the measurement error for the DRN of subject 3, before accounting for frame-to-frame variation, would therefore be equal to the sum of the average intercept value, the average individual deviation and the average regional deviation, but no further adjustment would be made for the particular DRN TAC recorded for subject 3 specifically.

For differences in the standard deviation of the measurement error, *σ*, across time, i.e. between the different frames within the measured TACs, we needed to select an appropriate weighting function, as a function of the measurement error. The measurement error should, in theory, be a function of the duration of each frame and the radioactivity counts observed, however there is no generally agreed-upon method by which to quantify noise in PET images. As such, many different weighting schemes exist (Muzic and Christian, 2006; Normandin et al., 2012; Thiele and Buchert, 2008; Yaqub et al., 2006), whose performance can vary between different radioligands and model parameters (Thiele and Buchert, 2008). Instead of selecting any one particular function, we estimated a weighting function simultaneously within the multifactor model using a smooth function over time *f*(*t*) to describe the standard deviation of the measurement error. For this, we used a penalised regression spline using a thin plate regression spline basis with 8 basis functions, implemented using the *brms* R package (Bürkner, 2017).

With all these considered, the model is described, as above with a global mean intercept (*α*), a covariate vector (*β*) multiplied with a covariate matrix (*X*), and an additive sequence of residuals for each of the separate hierarchies across individuals (*τ*_[*j*]_), across regions (*υ*_[*k*]_) and for the interaction of regions and individuals, i.e. individual TACs (*ϕ*_[*j,k*]_). For the measurement error, we include an additional smooth function over time (*f*(*t*)), indexed by frame *i*.


$$\begin{array}{llllll} \log\left(K_{1[j,k]}\right)=\overset{\text{Intercept}\;}{\overset{︷}{\alpha_{K_{1}}}} & +\overset{\text{Covariates}\;}{\overset{︷}{X_{K_{1}}\beta_{K_{1}}}} & +\overset{\text{Individual}\;}{\overset{︷}{\tau_{K_{1}[j]}}} & \overset{\text{Region}\;}{\overset{︷}{v_{V_{\text{ND}}}[k]}} & \overset{\text{TAC}}{\overset{︷}{\phi_{K_{1}[j,k]}}} & \overset{\text{Smooth}\;\text{function}}{\overset{︷}{}}\\ \log\left(V_{\text{ND}[j,k]}\right)=\alpha_{V_{\text{ND}}} & +{\text{X}}_{V_{\text{ND}}}\beta_{V_{\text{ND}}} & +\tau_{V_{\text{ND}}[j]} & +v_{V_{\text{ND}}}{}_{\left[k\right]} & +\phi_{V_{\text{ND}}[j,k]} & \\ \log\left(BP_{\text{ND}[j,k]}\right)=\alpha_{BP_{\text{ND}}} & +{\text{X}}_{BP_{\text{ND}}}\beta_{BP_{\text{ND}}} & +\tau_{BP_{\text{ND}}[j]} & & +\phi_{BP_{\text{ND}}[j,k]} & \\ \log\left(k_{4[j,k]}\right)=\alpha_{k_{4}} & & +\tau_{k_{4}[j]} & +v_{k_{4}[k]} & +\phi_{k_{4}[j,k]} & \\ \log\left(v_{\text{B}[j,k]}\right)=\alpha_{v_{\text{B}}} & +{\text{X}}_{v_{\text{B}}}\beta_{v_{\text{B}}} & +\tau_{v_{\text{B}}}{}_{\left[j\right]} & +v_{v_{\text{B}}[k]} & & \\ \log\left(\sigma_{\left[i,j,k\right]}\right)=\alpha_{\sigma } & +{\text{X}}_{\sigma }\beta_{\sigma } & +\tau_{\sigma [j]} & +v_{\sigma [k]} & & +f\left(t_{\left[i\right]}\right) \end{array}$$

$$\left[\begin{array}{c} \tau_{K_{1}}\\ \tau_{V_{\text{ND}}}\\ \tau_{BP_{\text{ND}}}\\ \tau_{k_{4}} \end{array}\right]\sim \text{MVNormal}\left(\left[\begin{array}{l} 0\\ 0\\ 0\\ 0 \end{array}\right],\;\Sigma_{\text{Subject}}\right)$$

$$\tau_{v_{\text{B}}}\sim \text{Normal}\left(0,\;\sigma_{v_{\text{B}},\text{Subject}}^{2}\right)$$

$$\tau_{\sigma }\sim \text{Normal}\left(0,\;\sigma_{\sigma ,\text{Subject}}^{2}\right)$$

$$\left[\begin{array}{c} v_{V_{\text{ND}}}\\ v_{k_{4}} \end{array}\right]\sim \text{MVNormal}\left(\left[\begin{array}{l} 0\\ 0 \end{array}\right],\;\Sigma_{\text{Region}}\right)$$

$$v_{v_{\text{B}}}\sim \text{Normal}\left(0,\;\sigma_{v_{\text{B}},\text{Region}}^{2}\right)$$

$$v_{\sigma }\sim \text{Normal}\left(0,\;\sigma_{\sigma ,\text{Region}}^{2}\right)$$

$$\left[\begin{array}{c} \phi_{K_{1}}\\ \phi_{V_{\text{ND}}}\\ \phi_{BP_{\text{ND}}}\\ \phi_{k_{4}} \end{array}\right]\sim \text{MVNormal}\left(\left[\begin{array}{l} 0\\ 0\\ 0\\ 0 \end{array}\right],\;\Sigma_{\text{TAC}}\right)$$

Predictors are included within the covariate matrices, including age, sex and patient group for pharmacokinetic parameters. Different parameters can have different sets of parameters: for instance, we might reasonably expect *K*_1_ to differ by age, but not *k*_4_. Model comparison methods, such as the LOOIC (leave one out information criterion) (Vehtari et al., 2017) are useful for evaluating whether additional predictors improve the performance of the model, for instance, to evaluate whether the addition of patient group as a predictor for *V*_ND_ improves the performance of the model.

### 3.4. Model fitting

The model was implemented using the STAN probabilistic programming language (Carpenter et al., 2017), which applies Hamiltonian Monte Carlo (HMC) for Markov Chain Monte Carlo (MCMC) simulation (Betancourt, 2018), using CmdStan v2.26.1, rstan 2.21.2 and *brms* 2.15.0 (Bürkner, 2017).

In the simulations, SiMBA was found not to converge in approximately 10% of the datasets. In all cases, this could be resolved by rerunning the model on the same data, but using a different random seed (i.e. by resetting the random number generator to a new state). Convergence was defined as there being no single parameter estimated by the model with an Rhat (Vehtari et al., 2021) value above 1.25, and no more than 2% of the parameters having an Rhat value above 1.05.

#### 3.4.1. Prior specification

In the definition of priors, our goal was not to greatly inform the model, but rather to exclude domains of parameter space which could *a priori* be deemed as extremely unlikely based on domain knowledge. For instance, the likelihood of several-fold inter-regional variability in *V*_ND_ or *k*_4_ can be rejected before seeing any data based on what these quantities represent. The primary goal of the priors, rather, was to restrict our model to sensible ranges of parameter space, and to equip our model with a skepticism for extreme outcomes.

For the distributional definitions of priors, we used normal distributions for most parameters, and student t distributions with 3 degrees of freedom when fatter tails (i.e. greater leptokurtosis) were required. Moderately informative priors were specified for the global intercept (*α*) terms to ensure that the model fitting procedure initialises in approximately the correct neighbourhood of the posterior. This can be justified because these values can easily be approximated from previous studies.

Zero-centred regularising priors were defined over the standard deviation of the pooled effects of subject, region and TAC, with progressively smaller standard deviation, owing to the expected decreasing magnitudes of these differences. This has the effect of informing our model *a priori* that no variation at all in the outcomes across the relevant hierarchy is the most likely outcome; and that larger values of the variance should be treated with an increasing degree of skepticism. This implies that for parameters such as *V*_ND_ and *ν*_B_, which are typically assumed equal between regions within individuals, our model is encouraged to comply with this assumption, but that deviations from this simplistic assumption are also allowed. These assumptions, however, are almost certainly oversimplifications: there is regional variation in the density of brain vasculature (i.e. affecting *ν*_B_) (Huck et al., 2019), and regional variation in *V*_ND_ has also been reported (Rossano et al., 2019).

LKJ priors (Lewandowski et al., 2009) were defined for the correlation matrices for each of these three multivariate normal distributions, with *η* = 1 for the pooling across individuals, and *η* = 2 for the pooling across regions and TACs, implying less and greater skepticism for extreme correlations respectively. Regularising priors were also defined for all covariates, including the unpooled regional differences in *K*_1_ and *BP*_ND_. For a more detailed description of the prior distributions, see the Supplementary Materials S1.

#### 3.4.2. Model comparison and diagnostics

To evaluate model performance, we made use of the *loo* package in R (Vehtari et al., 2020), which implements Pareto smoothed importance sampling (PSIS) leave-one-out cross-validation to derive the LOOIC (Vehtari et al., 2017). Information criteria are measures of predictive accuracy which are commonly used to assess whether the addition of greater complexity to a model improves or impedes the model’s predictive performance. Information criteria are measures of the expected log pointwise predictive density (ELPPD), often expressed on the deviance scale (i.e. −2 × ELPPD). The ELPPD is calculated by subtracting a penalty term for model complexity from the log pointwise predictive density (LPPD, also called the log likelihood).

The penalty term for model complexity is equal to the difference between the LPPD and the ELPPD, measuring the degree to which the prediction of future data is worse compared to the observed data. For a more flexible model, there is a greater risk of overfitting, and hence the prediction of future data is less accurate, and so this can be thought of as an overfitting penalty (Gelman et al., 2014; McElreath, 2016). In the Akaike Information Criterion (AIC), this penalty term is simply equal to the total number of fitted parameters. However, for models with informative priors or hierarchical structure, the overfitting risk does not scale in the same way with the number of parameters. In these cases, the number of parameters is replaced with a data-based bias correction, which is often referred to as the *effective* number of parameters (*p*_eff_) by analogy to the AIC. This term is dependent on the degree of constraint imposed on the estimation of the parameter. For instance, using uniform priors between (−∞ ∶ ∞), *p*_eff_ reduces to the total number of parameters. However for a highly informative prior, or in the context of hierarchical structure, *p*_eff_ can be considerably less than the total number of estimated parameters.

In hierarchical modelling, partial pooling is meant to establish a suitable compromise between no pooling and complete pooling of estimates, such that estimates are shrunk towards a global mean. When the precision of individual estimates is very poor relative to the group variance, the partial pooling approaches complete pooling, and *p*_eff_ is low. On the other hand, when the precision of individual estimates is high relative to the variance, then the partial pooling approaches no pooling, and *p*_eff_ is high. For more details see Gelman et al. (2014) and Vehtari et al. (2017). We make use of p_eff_ as an estimate of the complexity and the degree to which overfitting risk is reduced using our model relative to the traditional approach.

### 3.5. Data and code availability statement

The R and STAN code used to apply this method are provided in an open repository (https://github.com/mathesong/SiMBA_Materials), including a sample simulated dataset. The measured data used in the application section is drawn from previous studies (Chen et al., 2019).

## 4. Simulations

For the purpose of assessing the measurement properties of this approach, we generated simulated datasets to compare the performance of the proposed methodology to that of the conventional approach.

Data were simulated in order to resemble the true PET data described in the next section, using the [^11^C]WAY100635 radiotracer. We extracted the posterior mean values for all estimated population parameters and used these as the “ground truth” to generate realistic parameter values. For variation across individuals and TACs (i.e. Region × Individuals), we sampled individual new parameter values from the univariate and multivariate normal distributions, while for variation across regions, we made use of the posterior mean values. In this way, we simulate from the same set of regions, but in a new set of individuals, with a new set of individual variations at the regional level. The simulation parameters are described in Supplementary Materials S2.

TACs were simulated using the two-tissue compartment model using these parameter values, with blood data randomly sampled from individuals (with replacement) from the measured data. Measurement error was added to the simulated curves using a normal distribution with mean 0, and the standard deviation determined in a similar manner as for the pharmacokinetic parameters, i.e. using the posterior means for regional differences, but sampling individual differences from the univariate normal distribution. As before, this has the effect of simulating data from the same set of regions, but in a new set of individuals. To this, we added the global mean value of the SD of the measurement error, for which we used 10% of the mean TAC value. Rather than using the value estimated from the data, we opted to select a value which facilitates comparison with other data sets, and which more closely resembles a worst-case scenario. This value is approximately double that estimated from the measured PET data. Finally, we also added the posterior mean value of the smooth function for each time point within each TAC.

In order to evaluate the sensitivity and specificity of the approach, we tested for group differences. To evaluate power, we simulated two groups, with a true global (i.e., across all 9 regions) group difference of 20% in *BP*_ND_ (i.e., Δlog(*BP*_ND_) = 0.182).

Based on the mean posterior standard deviation of *BP*_ND_ across individuals in the sample, this corresponds with a moderate effect size (Cohen’s d = 0.55).

To evaluate the potential for false positives, we also tested for group differences in simulated data sets for which there were no differences.

No additional covariates, such as age or sex, were included, other than that of group membership.

We simulated datasets with group sizes of multiples of 10 between 10 and 100 (i.e., for a group size of 20, there are 20 controls and 20 patients, and therefore 40 individuals included in the study). For the NLS models, we generated 1000 simulated studies for each condition, i.e. for groups of 50, this results in a number of TACs of 1000 × 2 conditions (group differences vs no group differences) × 50 individuals × 2 groups × 9 regions. For the estimation of NLS parameters, each TAC was fitted 10 times with randomly sampled starting parameters and the best fit was selected using the *nls.multstart* package (Padfield and Matheson, 2018), to ensure that fits were optimal. In all cases, outcome parameters were calculated directly using the rate constants, and not indirectly using a reference tissue, i.e.$BP_{\text{ND}}=\frac{k_{3}}{k_{4}}$. It should be noted that the calculation of *BP*_ND_ in this manner is not a recommended practice using NLS, as it is known to be prone to error (Parsey et al., 2000; Slifstein and Laruelle, 2001). However, as we show below, direct estimation of *BP*_P_ is more accurate, and can be considered a better index of potential performance of NLS rather than *BP*_ND_.

For SiMBA, fitting the model to so many datasets would have incurred a very large computational burden owing to the greater computational requirements. Instead, we limited sample sizes to group sizes of 10, 20 and 50, and generated only 50 simulated studies for each condition. Applying the model to these datasets resulted in approximately 1.5 core years of processing.

### 4.1. Comparison of power for detecting group differences

Using the simulated data described above, we performed inference on group differences to determine the power or sensitivity, i.e., true positives, and specificity, i.e., false positives, of the model.

For inference for the NLS results, we performed both t-tests and linear mixed effects (LME) modelled using the generated outcome parameters after being transformed to their natural logarithms. Welch’s t-tests were fit for each region separately, while the LME model was applied across all regions, with fixed effects for region and for group membership, and a random intercept for individuals. These are both common strategies employed in clinical PET studies, which are used as a basis for comparison. Although for global differences the LME model is obviously more appropriate, t-tests are often employed in PET studies, even when differences are global: our intent was not to compare these two approaches with one another, but to provide an appropriate baseline comparison for the SiMBA model. P-values for the LME were calculated using the lmerTest package (Kuznetsova et al., 2017).

For the hierarchical Bayesian model, binary inferences were determined by assessing whether the 95% credible interval of the posterior estimate of the group difference included or excluded zero. Due to the small sample size, we fit logspline density functions (Stone et al., 1997) to both the upper and lower bounds of the 95% credible intervals across simulations, and estimated the proportion of the distributions for which the estimates would not include zero using their cumulative density functions. The logspline fits were visually assessed, and estimates were closely aligned with the empirical estimates (Supplementary Materials S3). For this reason, we have included 95% confidence intervals around the estimated power for SiMBA. Furthermore, in order to confirm that the different simulated datasets did not induce any bias, we also performed all NLS analyses using the same data to which SiMBA was applied, using both empirical and logspline estimation, which produced very similar results (Supplementary Materials S4). We calculated 95% confidence intervals for the power using bootstrap resampling, i.e., by repeating the logspline procedure for 1000 samples of the 50 outcomes sampled with replacement.

**Fig. 3.**
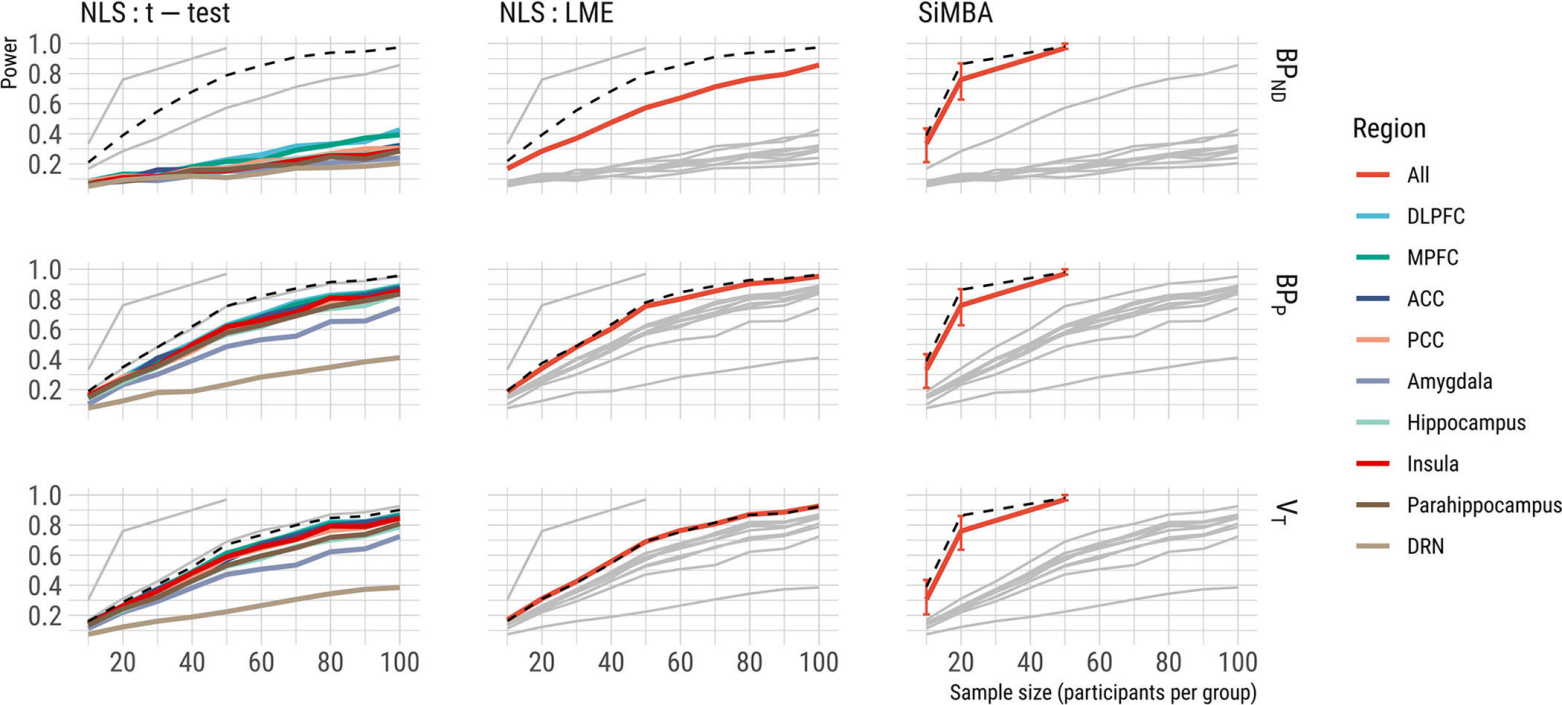
Power is shown here for each method for a true effect size of 20% in *BP*_ND_ (Cohen’s d = 0.55) for different sample sizes. The results of the other methods are shown in grey to assist with comparison. Dashed lines represent the power of each method when applied to the true simulated values for comparison, i.e. incorporating sampling variation, but without any error in the parameter estimation. Regional abbreviations are as follows: DLPFC is dorsolateral prefrontal cortex, MPFC is medial prefrontal cortex, ACC is anterior cingulate cortex, PCC is posterior cingulate cortex, and DRN is dorsal raphe nucleus.

**Fig. 4.**
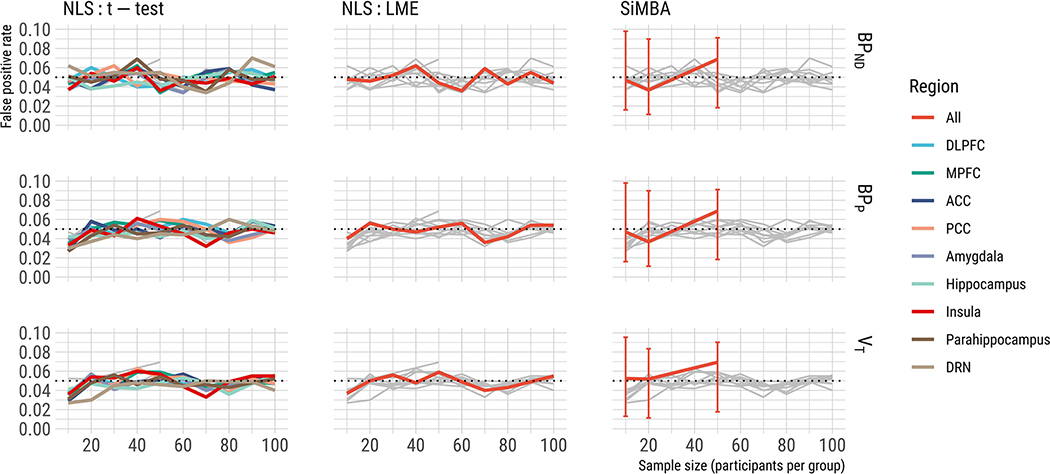
False positive rate is shown here for each method for a true effect size of 0% in *BP*_ND_ for different sample sizes. Dotted lines represent a 5% false positive rate for comparison. The results of the other methods are shown in grey to assist with comparison. Regional abbreviations are as follows: DLPFC is dorsolateral prefrontal cortex, MPFC is medial prefrontal cortex, ACC is anterior cingulate cortex, PCC is posterior cingulate cortex, and DRN is dorsal raphe nucleus.

As shown in Fig. 3, we show that the LME model exhibits greater power compared to regional t-tests as expected, and we show that the SiMBA model exhibits substantially increased power relative to both of these methods. This suggests that SiMBA demonstrates greater sensitivity to detect true differences between groups for the same sample size, exhibiting power equivalent to sample sizes of approximately double using NLS estimation and LME. We also show that no models exhibit a false positive rate that is significantly or substantially differ from 5% (Fig. 4). Taken together, this suggests that SiMBA is more sensitive than the traditional NLS methods, without sacrificing specificity

Although LME applied to *BP*_ND_ shows poor performance in Fig. 3, likely owing to poor estimation, LME applied to *BP*_P_ exhibits similar or only marginally reduced power compared to when LME is applied to the true values. This supports direct estimation of BP_P_ as a good index of specific binding using NLS.

### 4.2. Effect size estimation

**Fig. 5.**
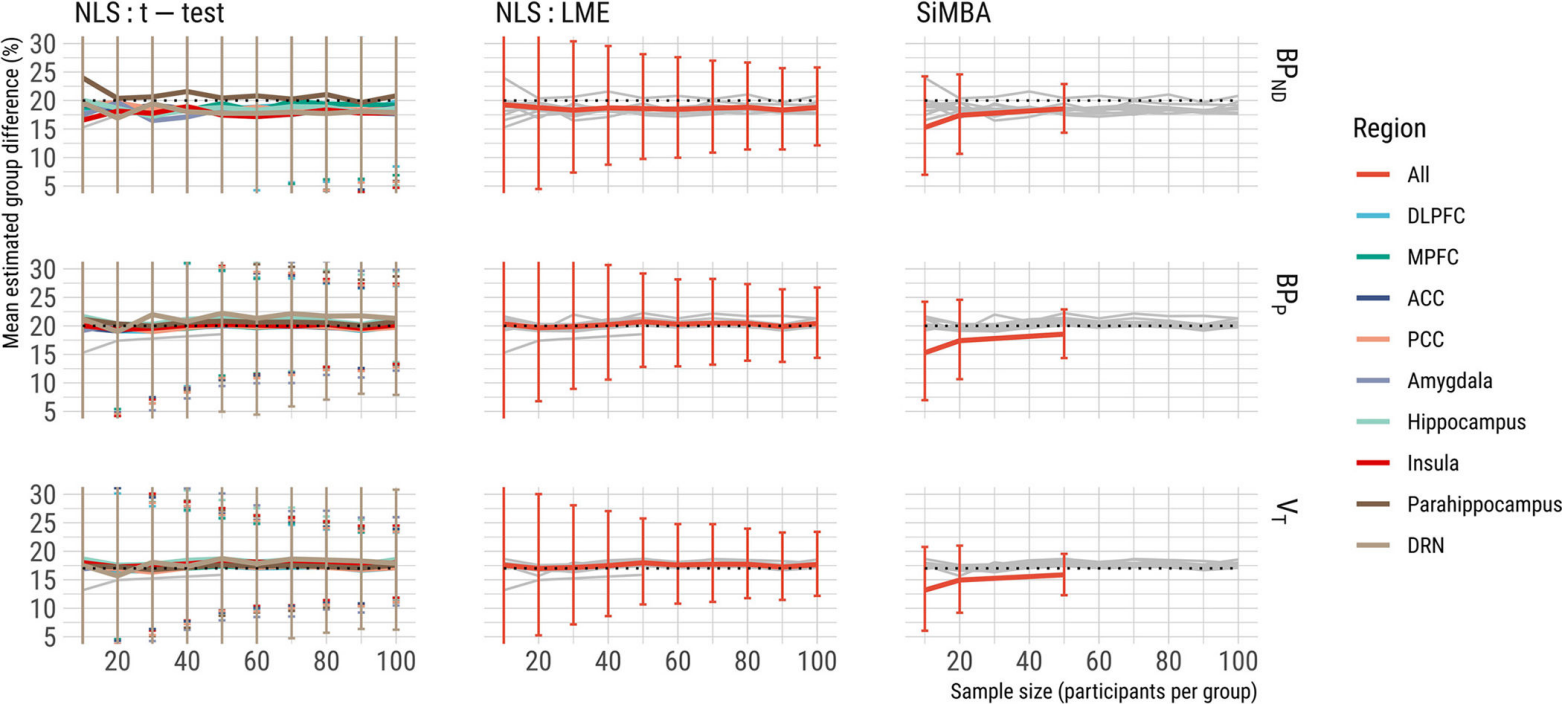
Mean estimated group differences shown for all methods. Error bars represent the standard deviation across simulations for each method. A full comparison of the standard deviation of the estimates is presented in Supplementary Materials S5. The results of the other methods are shown in grey to assist with comparison. The y axis has been truncated to emphasise the estimation bias as well as the differences in SD between LME and SiMBA. Regional abbreviations are as follows: DLPFC is dorsolateral prefrontal cortex, MPFC is medial prefrontal cortex, ACC is anterior cingulate cortex, PCC is posterior cingulate cortex, and DRN is dorsal raphe nucleus.

A secondary objective of this study was to assess how well this new approach estimates the magnitude of the “true” effect. In the simulations for which the true group difference in *BP*_ND_ was equal to 20%, we assessed the mean of the model estimates of the group differences and their standard deviation across the simulations. The results are shown in Fig. 5.

We observe a small degree of negative bias in the model estimates with SiMBA for all outcome parameters and all sample sizes, which is greater for smaller sample sizes, and smaller for larger sample sizes. This was not present for *BP*_P_ or V_T_ in the NLS estimates, although there was a small negative bias in *BP*_ND_ estimates.

Beside the observed bias, we also compared the standard deviation of estimates of group difference between simulations with each method (error bars in Fig. 5, and in Supplementary Materials S5). We show reduced SD of group difference estimates between simulated datasets for the LME compared to the t-tests, as well as improved consistency of estimates using SiMBA compared to the NLS approaches, comparable to that observed in sample sizes of approximately four times larger using LME.

The observed bias in SiMBA estimates is small with respect to the standard deviation of estimates across simulated datasets: 53% for n=10, 36% for n=20 and 33% for n=50. This means that, given infinite repetitions of a study with sample sizes of n=10, estimated effect sizes will be still be higher than the true value in 30% of these repetitions. As such, this bias is practically negligible in an applied context.

### 4.3. Outcome parameter estimation

We also evaluated the accuracy of binding estimates for individual TACs, rather than for population group differences. We fit the NLS model to a new set of 1000 simulated TACs from each region. For SiMBA, we evaluated its accuracy in simulations of studies comprised of different sample sizes: this is because SiMBA utilises the total sample to estimate binding within each individual TAC, and its performance ought therefore to improve with larger sample sizes. For this reason, we extracted model estimates of individual binding values from the first 1000 individuals from the SiMBA simulations with n=10, n=20 and n=50 in which there were no differences between groups.

**Fig. 6.**
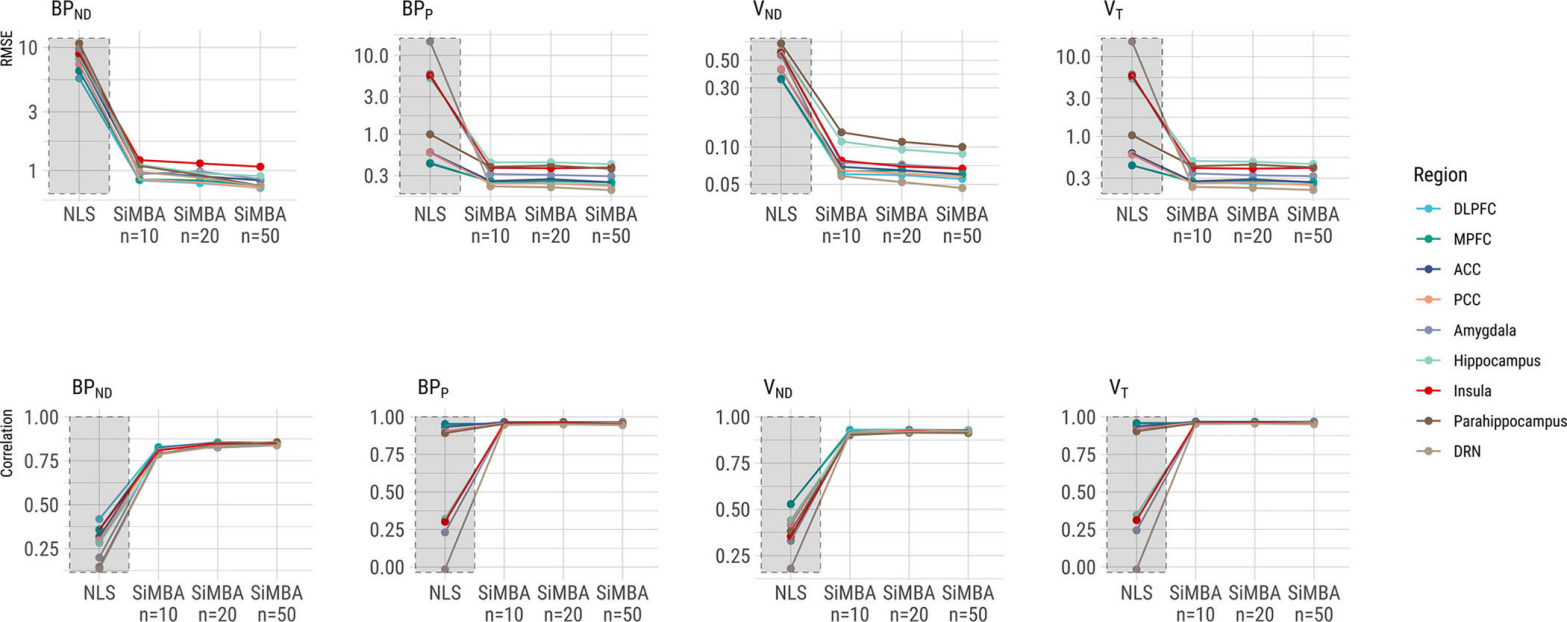
Correspondence between individual true binding outcome values and estimated outcomes. RMSE represents the root-mean-square error, a measure of absolute deviation from the true values. Correlation is the Pearson’s r correlation, used as a measure of relative correspondence between the true and measured values. The n represents the number of participants per group, i.e. *n* = 10 corresponds to a total sample size of *n* = 20. Regional abbreviations are as follows: DLPFC is dorsolateral prefrontal cortex, MPFC is medial prefrontal cortex, ACC is anterior cingulate cortex, PCC is posterior cingulate cortex, and DRN is dorsal raphe nucleus.

We observe greater correspondence between true simulated binding values and estimated values using SiMBA compared to the NLS estimates, in terms of both the root-mean-squared error (RMSE) as a measure of absolute accuracy, as well Pearson’s r as a measure of the relative accuracy. The results are presented in Fig. 6.

Differences between NLS and SiMBA were most pronounced for *BP*_ND_ and *V*_ND_. This is likely attributable to the use of direct estimation using NLS (Parsey et al., 2000; Slifstein and Laruelle, 2001). However, even for *V*_T_, SiMBA exhibits marked improvements in estimation accuracy.

We also show that with larger sample sizes, the accuracy of the MCMC increases, however these increases are subtle. This suggests that SiMBA improves accuracy even in small sample sizes.

### 4.4. Sensitivity to measurement error

Lastly, we assessed the sensitivity of the SiMBA model to varying degrees of measurement error. To this end, we simulated sets of 50 studies of simulated data as above with *n* = 10 per group and group differences of 20%, but with the mean standard deviation of the measurement error set as equal to 2.5%, 5%, 10% and 20% of the mean TAC value. With increasing measurement error, the estimated power decreased (from 38% to 22%), and we observed increases in both the standard deviation of estimated group differences between the simulated studies, as well as the mean standard error of estimated group differences within simulated studies (Supplementary Materials S6). It is notable that even with 20% measurement error, the estimated power for SiMBA was still numerically higher compared to the NLS outcomes in Fig. 3 with half the measurement error.

### 4.5. Multivariate considerations

In Fig. 3, it is also apparent that SiMBA exhibits even higher power than the t-tests or LME models do when applied using the true simulated values of binding estimates. We considered this worthy of extra investigation to make sure that it was not indicative of pathological behaviour of the model - despite the lack of increase in the false positive rate. We tested two potential reasons for how this could occur: firstly, we tested whether this could be related to the multivariate nature of SiMBA, which allows the model to pool information across the different pharmacokinetic parameters through their intercorrelations, despite not testing for between-group differences in the other parameters. Secondly, we tested whether the improved power of SiMBA might be attributable to over-regularisation of *BP*_ND_ values, i.e. excessive shrinkage of the between-subject variation. While the over-regularisation hypothesis would imply that the improved power is due to a pathological model specification, i.e. a “bug,” the multivariate hypothesis implies that it is advantageous, i.e. a “feature.”

To test for over-regularisation, we evaluated the distribution of standardised mean difference values of *BP*_ND_ in the simulated studies, i.e. Cohen’s d. If the model was excessively shrinking *BP*_ND_ values towards the mean, the separation between groups would be artificially increased and the Cohen’s d value would be inflated. When examining the data, however, we did not observe any inflation of Cohen’s d values: rather we observed close alignment between estimated values and the true standardised differences, and even a tendency for underestimation: for the true Cohen’s d = 0.55, the mean estimates were similar for each sample size. For n=10, mean d = 0.46 (95% CI: 0.04 – 1.18); n=20, d=0.52 (0.14 – 0.97); and for n=50, d=0.54 (0.28 – 0.73). We therefore reject this hypothesis.

To test for whether this is an effect of the multivariate model specification, we used the simulated datasets to which SiMBA was applied with n=20 and a true difference of 20% in *BP*_ND_ (i.e. 0.182 in log(*BP*_ND_)). Firstly, we fit a modified version of SiMBA using univariate normal distributions in place of multivariate normal distributions with all the same priors. While the original multivariate SiMBA model showed 76.0% power for *BP*_ND_ (95% CI: 63.7 – 85.4%), the power of the univariate SiMBA model was reduced to 24.5% (95% CI: 14.8 – 36.4%). Furthermore, compared to the univariate SiMBA, the multivariate SiMBA exhibited less bias of the mean group difference (uni: 0.13, multi: 0.16), lower standard deviation of estimates between simulations (uni: 0.075, multi: 0.059), and lower standard error of group difference estimates (uni: 0.090, multi: 0.061). Secondly, we also fit univariate and multivariate multifactor Bayesian models, using the same priors, to the true values from the simulations, i.e. without modelling the TACs. In a similar fashion, the multivariate Bayesian analysis yields higher power (86.4%, 95% CI: 76.0 – 93.6%, as shown in Fig. 3) than the univariate analysis (20.6%, 95% CI: 11.5 – 30.1), as well as less bias of the mean (uni: 0.12, multi: 0.17), lower standard deviation (uni: 0.064, multi: 0.057), and lower standard error (uni: 0.090, multi: 0.056). We therefore conclude that the even better performance of SiMBA compared to the true values used in the conventional manner is explained by its exploiting the estimated correlations between parameters to better inform its inferences, and is not indicative of any issues with the model definition.

Lastly, we tested whether the observed correlations might be artefactual, i.e. induced by estimation inaccuracies rather than resulting from true correlations. To this end, we simulated datasets with n=20 in each group and with the same variances as the simulated data, but with no correlation between the simulated parameters in the individual or TAC hierarchies. At the TAC level, all bivariate correlations were centred around zero. At the individual level, all but one of the 6 correlations were centred around 0. The only exception was that of the correlation between *BP*_ND_ and *V*_ND_, which even showed a tendency to be stronger than estimated in the datasets with true correlations. Nevertheless, despite the measurement error of the simulated data being more than twice as large as in the original data, eleven of the twelve tested associations were centred around zero, suggesting that the correlations estimated from the original data are unlikely to be completely artefactual, although they may be partially induced by estimation inaccuracies. For more information, see Supplementary Materials S7.

## 5. Application in measured data

The serotonin 1A receptor (5-HT_1A_R) is thought to play an important role in major depressive disorder (MDD) (Kaufman et al., 2016; Shrestha et al., 2012), as well as its treatment (Blier et al., 1987; Gray et al., 2013). The receptor itself functions both as an autoreceptor in the dorsal raphe nucleus (DRN), reducing the global release of serotonin in the brain, and as a postsynaptic heteroreceptor in projection regions of the brain. [^11^C]WAY100635 is the most commonly used PET tracer to image this receptor in the brain, however studies of MDD with [^11^C]WAY100635 have been complicated by several methodological considerations, primarily involving the inadequacy of the cerebellum as a reference region for the indirect calculation of *BP*_P_ or *BP*_ND_ (Hirvonen et al., 2007; Shrestha et al., 2012). Our data consisted of PET data measured using [^11^C]WAY100635, acquired from 97 individuals. These data consist of 56 healthy controls and 41 patients with MDD, of whom 21 had recently been exposed to antidepressants (AE), while 20 were not recently medicated (NRM) (Parsey et al., 2010). All subjects gave written informed consent prior to participation, and all studies were approved by the regional ethics committees.

PET measurements were collected for 115 minutes, with 20 frames of duration:$3\times \frac{1}{3}$, 3 × 1, 3 × 2, 2 × 5, 9 × 10 min. We applied the model to TAC data extracted from 9 regions. Fourteen individuals were missing one or more frames due to technical issues, resulting in a total of 17,298 observations.

Additional covariates for the pharmacokinetic parameters include age and sex for *K*_1_, *V*_ND_ and *BP*_ND_, and a region × diagnosis interaction for *BP*_ND_ to account for potential regional differences. For the measurement error (*σ*), average region size as well as injected radioactivity were included as covariates, both of which were first log-transformed and then centred.

**Fig. 7.**
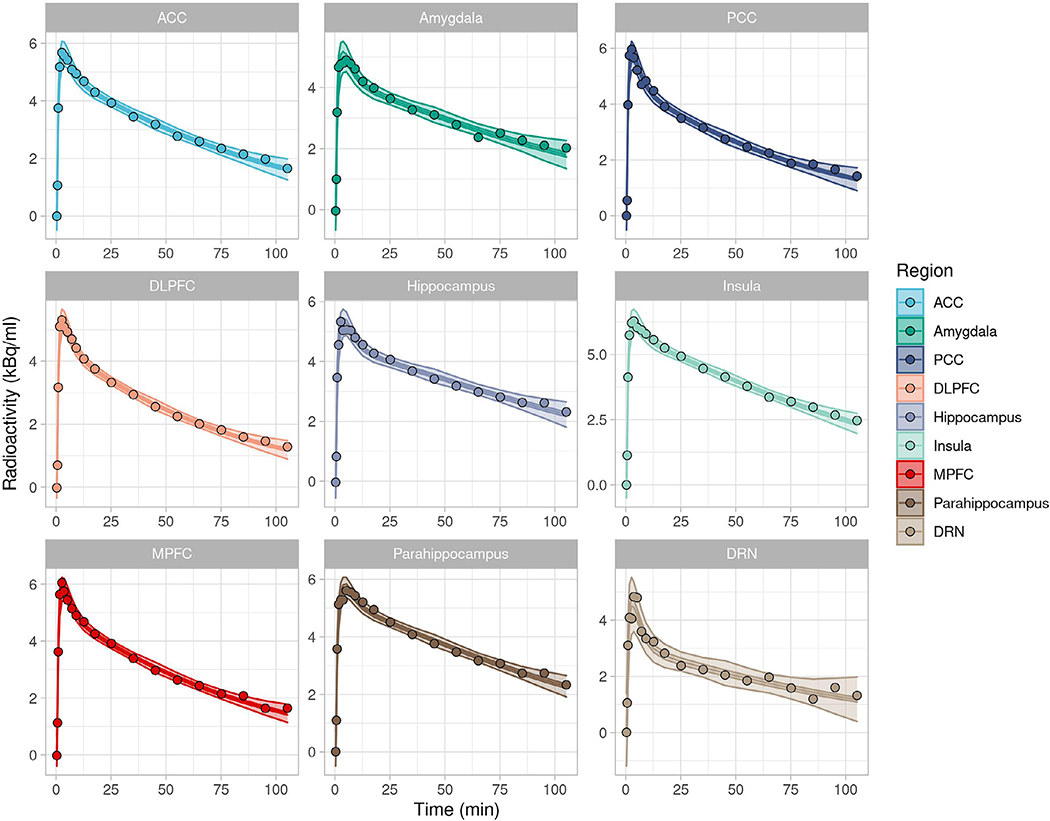
Representative TACs for one individual. Shown are the data points with mean posterior fitted line, surrounded by the 95% credible interval, which itself is surrounded by the 95% prediction interval. The credible intervals enclose the region in which 95% of the posterior probability is located for where the predicted curve lies, while the prediction intervals enclose the region in which the model assigns a 95% probability that the data will be observed.

SiMBA was applied to this data as described, and model estimates for the TACs of a randomly selected individual measurement are presented in Fig. 7. Using LOOCV, the effective number of parameters (*p*_*eff*_) was estimated to be 1,879.5, corresponding to 2.2 effective parameters per TAC. This can be contrasted with the 5 parameters per TAC that must be estimated when using the NLS approach. It should also be noted that this comparison is only approximate, as the number of parameters estimated by the SiMBA model includes not only the PK parameters, but also measurement error and all the covariates for all the parameters. The actual number of parameters estimated in both cases are 4240 for SiMBA, and 4365 for NLS. This number is lower for SiMBA is because of our decision not to estimate additional Region × Individual variation in *ν*_B_, i.e.$\phi_{v_{\text{B}}}$, which would have accounted for another 873 parameters.

The inferences for the covariates (excluding regional differences) are presented in Table. 1. For each variable, we present the estimate and its 89% credible intervals, following the recommendations of (McElreath, 2016). We also present the directional probability (Pd), indicating the posterior probability that the estimate is in the direction of the posterior median: as such this value lies between 0.5 and 1) (Makowski et al., 2019).

In frequentist statistics, the model estimates the probability of the data conditional upon different values of the estimated parameters, *P*(*Data*|*θ*). In contrast, the posterior probability distribution in Bayesian statistics represents our model’s updated degree of certainty, and uncertainty, regarding potential values of the model parameters, conditional upon the prior and the data (i.e. the likelihood), *P*(*θ*|*Data, Prior*), and can be interpreted as such. Hence we can interpret the results of the model as that, given the model, the prior and the data, there is a high probability that *K*_1_ values decrease with age, and are lower in males compared to females. Regarding the effects of MDD and antidepressant medication, there is an 89% probability that [^11^C]WAY100635 *BP*_ND_ is decreased following exposure to antidepressant medication in the DRN, and a 94% probability that it is increased in the same region in depressed patients who have not recently been medicated. In both cases, the mean parameter estimate is indicative of differences of a very small magnitude (4.4% and 5.5% changes in *BP*_ND_ respectively). However, estimated differences in the DRN are over twice as large as in any other region in both groups of patients, and the probability of changes in the other regions is low. These results correspond with empirical research, suggesting that depression may be associated with increased 5-HT_1A_ autoreceptor function in the DRN, which reduces serotonin release, which is reversed following exposure to antidepressant medication (Gray et al., 2013; Richardson-Jones et al., 2010). While previous PET imaging studies have observed increases in [^11^C]WAY100635 BP_F_ in unmedicated patients compared to controls (Parsey et al., 2010; 2006), these studies have not observed differences in *BP*_ND_, and they have made use of indirect quantification using the cerebellum as an imperfect reference region rather than estimating the binding potential directly.

## 6. Discussion

In this study, we demonstrate a new approach for fitting PET pharmacokinetic models to TAC data, using a Bayesian hierarchical multifactor model, which allows the model not only to borrow strength across both regions and individuals, but also simultaneously to perform statistical inference. This functions to incorporate all uncertainty of the estimates as well as gaining strength from intercorrelations between pharmacokinetic parameters. Using simulations, we demonstrate that this approach substantially improves the power to detect a true difference between groups - even beyond that which is possible using the true simulated values of the binding outcomes using conventional analysis - without affecting the false positive rate. We also show that, while this approach exhibits a small degree of bias, it is substantially more consistent in its estimation of effect sizes; and that it improves the estimation not only of population differences, but also of individual binding values. When applied to a real dataset, the model yields a good fit to TAC data, and parameter estimates which are biologically plausible. In sum, we believe that this approach presents a more efficient, accurate and robust method by which to perform PET kinetic modelling and analysis.

Making use of more data in a model is a commonly applied strategy for improving parameter estimation in PET modelling, either simultaneously (Endres et al., 2011; Ogden et al., 2015; Raylman et al., 1994; Slifstein et al., 2015) or by estimating one or more parameters in advance (Ichise et al., 2003; Logan et al., 1996; Wu and Carson, 2002). Partial pooling is a statistically principled technique which serves to establish the appropriate balance between estimating parameters independently, or as identical, and thereby optimises the pooling of information across the sample. Nonlinear partial pooling approaches have been applied in PET to estimate parent fraction concentrations (Varrone et al., 2020; Veronese et al., 2013), as well in modelling TACs either between individuals (Chen et al., 2019; Kågedal et al., 2012; van Rij et al., 2005; Syvänen et al., 2011) or between regions (Berges et al., 2013; Kågedal et al., 2015; Zamuner et al., 2002). The current study is the first, to our knowledge, in which PET TAC data has been modelled simultaneously using a multifactor model across both regions and individuals; both of which provide unique information with which the model can constrain and thereby improve estimation.

Reducing model complexity is the most common strategy for improving the robustness of PET kinetic models, but in the absence of partial pooling, this has always been accompanied by compromises and assumptions made at the level of the applied pharmacokinetic model. Here, estimating not only the pharmacokinetic parameters, but also the blood volume fraction, weighting function, and covariate parameters, the complexity of our model is reduced to the level of a one-tissue compartment (1TC) model estimated in the traditional manner — or even simpler than the 1TC if the blood volume fraction is also fitted (i.e. as a 3-parameter model). This reduced complexity requires the assumption that individuals and regions can be modelled as originating from common overarching population distributions. However this assumption is inherently reasonable in most applications of PET, as the same assumption is also usually made when performing statistical analysis using the parameters estimated with the conventional approach, for example when comparing groups. We show that this reduced complexity is not accompanied by compromises at the level of parameter estimation: rather we show that estimation of both group differences and even individual participants’ parameters are improved, in both large and small samples. This makes it possible to apply more complex pharmacokinetic models which yield more detailed information, with greater stability and identifiability of the estimated parameters. This is likely to be useful in numerous applications of PET neuroimaging. For instance, for kinetic modelling of the recently-developed radiotracer [^11^C]UCB-J, despite the 2TC model being favoured by model comparison, its estimates of V_T_ tend to be unstable: this has led to the 1TC being preferred in practice (Finnema et al., 2018). The current results suggest that SiMBA ought not only to stabilise the 2TC model, but also make it possible to reliably estimate *BP*_ND_ or *BP*_P_ directly without the use of a reference region. In theory, SiMBA should provide improved estimation compared to the traditional approach provided that the distributional assumptions are met, however the degree to which SiMBA exhibits improvements over the traditional approach will likely depend on interregional and interindividual variance, as well as the strength of correlations between regions and parameters.

It can be argued that this approach formalises many of the typical strategies which PET modellers usually make use of in a more rudimentary and *ad hoc* fashion. Modellers must be vigilant for cases in which the model fitting algorithm has fallen into a local minimum and produces an incorrect, and unlikely, set of outcomes. One common example is observing an unusually high V_T_ value originating from an uncharacteristically low *k*_4_ value, which is often caused by a small upward deviation in the TAC from one or more of the last frames of the measurement. In this case, PET modellers will usually check the fitted parameters for irregularities, and correct this by adjusting starting parameters, upper or lower limits, or choosing a more appropriate weighting function. In the case of hierarchical regression, individual estimates are shrunk towards the population mean value: this has the function of mathematically encoding a natural skepticism for extreme values in a statistically principled manner.

In our simulations *BP*_ND_ was only estimated using direct estimation for NLS, and not using indirect methods. This is not a recommended method for quantification of this parameter using NLS (Slifstein and Laruelle, 2001) owing to its poor stability. This is reflected in poor accuracy (Fig. 6), as well as low power (Fig. 3). Indirect quantification of *BP*_ND_ using [^11^C]WAY100635, using the cerebellum as a reference region, typically using the simplified reference tissue model (Lammertsma and Hume, 1996), is more common in practice, however this is not without issue owing to several troublesome properties of the cerebellum as a reference region for this tracer (Hirvonen et al., 2007; Parsey et al., 2000; Shrestha et al., 2012). Application of reference tissue modelling in our simulated data is problematic owing to the lack of regional correlation of the measurement error. On the other hand, we observe that *V*_T_ and *BP*_P_ estimated directly exhibit similar power using LME compared to the true values. This implies that there are no substantive improvements of statistical power possible for this application using univariate LME. SiMBA is only able to outperform this owing to its multivariate specification.

There are several important limitations to this approach. The most obvious limitation is that Bayesian modelling requires significant computational resources. On average, using 3 processors in parallel, the simulations took approximately 15 minutes per subject, i.e., 5 hours to fit the data from 20 individuals in the n=10 simulations, and 25 hours for the n=50 simulations. The simulations were also run with a relatively small number of iterations, so increasing the number of iterations to improve the estimation in real datasets would increase the computation time further in a linear fashion. For instance, when applying SiMBA to the real data in the Analysis section, we run approximately three times as many iterations. Secondly, SiMBA as it is currently implemented, is reliant on the parametric description of the arterial input function by a linear rise followed by a tri-exponential decay. The AIF of some tracers, however, cannot be described using this parameterisation, and therefore the model cannot, in its current form, be applied in these cases. We are hoping to develop an extension for this methodology which will be able to accommodate more complex AIF data.

A more general limitation of this approach is that it requires skills and expertise in Bayesian statistical modelling and the application of MCMC, but also for ongoing collaboration with domain experts. Optimal deployment of this approach should be accompanied by careful model specification and prior elicitation tailored to the specific application, involving ongoing collaboration between modellers/statisticians, clinicians and PET specialists to define principled priors, alongside iterative model development and checking in a Bayesian workflow (Betancourt, 2020b; Gelman et al., 2020). We would therefore strongly advise against the application of this model in a “default” manner. Optimal model specification and design is made simpler by the many recent advancements made in computational Bayesian methodology. We used STAN to apply MCMC, which itself applies an algorithm which is a variant of HMC. While HMC is renowned for being fast and efficient, it allows for the assessment of a host of diagnostics to identify degeneracies in the posterior distribution: in other words, it “fails loudly.” Recent advancements in Bayesian visualisation methodologies and tools also make it easier to quickly evaluate the model and its performance, and to identify insufficiencies in the model definition or estimation (Gabry et al., 2019). Additionally, the PSIS implemented in the loo package allows not only for model selection, but also model evaluation using Pareto k diagnostics, which provide an estimate of each observation’s influence on posterior distribution (Vehtari et al., 2020; 2017). This latter diagnostic makes it clear for which specific time points and in which specific individuals the model is performing most inadequately. For instance, this was helpful in identifying that regional variation in the blood volume fraction was necessary in our model.

An important advantage of this study is its implementation using R (R Core Team, 2022) and STAN (Carpenter et al., 2017). Both of these tools are open-source, and freely available. This makes it easier to apply this method in high-performance clusters, or through cloud computing infrastructure, and they can easily be incorporated into a Docker container for example (Boettiger, 2015). The R and STAN code used to apply this method are provided in an open repository (https://github.com/mathesong/SiMBA_Materials).

The potential value of SiMBA is not limited to prospective studies; it is also highly promising for the retrospective re-evaluation of already-collected data. Due to the high costs of PET, alongside the numerous other constraints for imaging psychiatric or neurological patients, most clinical PET datasets have been rather small. We anticipate that this method will make it possible to study more clinically-relevant research questions which could not previously be answered with sufficient power in these datasets, and thereby to improve the clinical relevance of PET imaging. However, the potential benefits of retrospective re-analysis of existing data is considerably augmented in the context of recent steps taken within the field to promote data sharing, as well as to harmonise data storage, reporting and analysis procedures (Knudsen et al., 2020; Norgaard et al., 2022). Combined with the pooling of smaller datasets from individual research centres, we anticipate that the potential for SiMBA to reveal new, clinically-relevant associations will be even greater.

The SiMBA approach is by no means limited to its current implementation, and we plan to extend it in several ways. As discussed, we intend to implement functionality to make it possible to use SiMBA when the AIF cannot be described by a tri-exponential function. We are also working on extending SiMBA to make use of plasma free fraction measurements to estimate *BP*_F_ rather than *BP*_ND_. Furthermore, we will soon be implementing functionality to incorporate the estimation of receptor occupancy parameters within the SiMBA model. In summary, we hope that the current implementation of SiMBA serves not as an end point, but as a point of departure for new possibilities.

## Supplementary Material

1

## Figures and Tables

**Table 1:** Mapping human errors to requirements engineering activities.

Covariate	Estimate (%)	L89	U89	Rhat	Pd	Visualisation

***K*_1_**						
Sex (Male - Female)	−4.6	−9.5	0.4	1.00	0.93	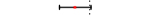
Age (per decade)	−4.1	−7.1	−1.1	1.01	0.99	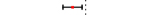
***V*_ND_**						
Sex (Male - Female)	1.0	−5.1	7.6	1.00	0.61	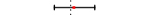
Age (per decade)	−3.5	−7.9	1.2	1.01	0.88	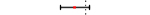
***BP*_ND_**						
Sex (Male - Female)	−0.4	−6.3	5.8	1.01	0.54	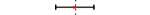
Age (per decade)	−0.6	−4.3	3.3	1.00	0.61	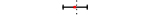
***BP*_ND_: Antidepressant-Exposed - Control**						
DLPFC	−1.1	−5.9	4.0	1.00	0.63	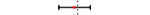
MPFC	−0.6	−5.5	4.6	1.00	0.57	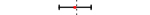
Hippocampus	0.9	−4.2	6.4	1.00	0.60	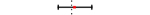
Amygdala	−0.4	−5.6	5.1	1.00	0.55	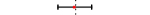
Parahippocampus	0.5	−4.8	5.7	1.00	0.57	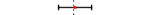
Insula	−1.8	−6.8	3.4	1.00	0.71	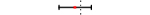
ACC	1.6	−3.4	7.0	1.00	0.70	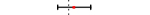
PCC	0.8	−4.3	6.1	1.00	0.60	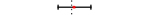
DRN	−4.4	−9.7	1.3	1.00	0.89	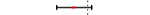
***BP*_ND_: Not Recently Medicated - Control**						
DLPFC	−1.1	−5.8	4.0	1.00	0.64	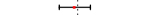
MPFC	−0.3	−5.2	4.7	1.00	0.54	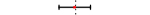
Hippocampus	−2.1	−7.0	3.3	1.00	0.74	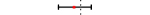
Amygdala	−2.0	−7.2	3.3	1.00	0.73	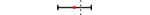
Parahippocampus	−0.5	−5.5	4.8	1.00	0.57	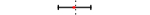
Insula	−0.5	−5.5	4.8	1.00	0.56	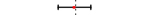
ACC	0.3	−4.8	5.6	1.00	0.54	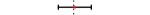
PCC	−0.3	−5.3	4.8	1.00	0.54	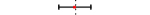
DRN	5.6	−0.2	12.0	1.00	0.94