# Supplementary material for: Simultaneous multifactor Bayesian analysis (SiMBA) of PET time activity curve data
Source: Neuroimage. Author manuscript; Available in PMC 2022 Sep 13. (PMC9470242; doi:10.1016/j.neuroimage.2022.119195)
Supplement: 1 [file NIHMS1812867-supplement-1.pdf]

Supplementary Materials

Contents

Supplementary Materials S1 : Prior Specification **2**

Global Intercepts 2

Individual effects 2

Regional effects 3

TAC effects 4

Smooth sigma function 4

Covariates 5

Analysis data 5

Supplementary Materials S2 : Simulated parameters **6**

Global Intercepts 6

Individual effects 6

Regional effects 6

TAC effects 7

Smooth sigma function 7

Supplementary Materials S3: Comparisons between power estimation approaches **9**

Supplementary Materials S4: Group difference properties in the same data **12**

Supplementary Materials S5 : SD of estimated mean differences by method **14**

Supplementary Materials S6: Sensitivity to measurement error **15**

Supplementary Materials S7: Application to data with no parameter intercorrelations **16**

Supplementary Materials S1 : Prior Specification

We made use of a multifactor model (Betancourt, 2021), with a global mean, together with partially pooled deviations from the global mean for each region across all individuals, and for each individual across all regions. We also added a final partially pooled parameter to accommodate any residual differences at the level of individual TACs after accounting for average regional and individual effects. We therefore anticipate that most of the variation in the pharmacokinetic parameters will be accounted for by individual differences and regional differences, and that TAC-level will be much smaller. For this reason, we defined wider zero-centred priors over the standard deviation across individuals and regions, but more constrainted priors over the standard deviation across TACs.

For the simulations, we did not include any covariates (other than region where we used unpooled effects for $\mathrm{BP}_{\mathrm{ND}}$ and K_1) for any parameters besides $\mathrm{BP}_{\mathrm{ND}}$, which was compared between the two groups.

Global Intercepts

Below are the priors defined for the global intercepts. Note that all priors are defined over the natural logarithms of the parameters.

$$
\begin{aligned}
\alpha_{K_1} &\sim \mathrm{Normal}(-2.5\ ,\ 0.25) \\
\alpha_{V_{\mathrm{ND}}} &\sim \mathrm{Normal}(-1.0\ ,\ 0.25) \\
\alpha_{BP_{\mathrm{ND}}} &\sim \mathrm{Normal}(\ \ 2.0\ ,\ 0.25) \\
\alpha_{k_4} &\sim \mathrm{Normal}(-4.0\ ,\ 0.25) \\
\\
\alpha_{\mathrm{v_B}} &\sim \mathrm{Normal}(-4.0\ ,\ 0.50) \\
\\
\alpha_{\sigma} &\sim \mathrm{Normal}(-5.0\ ,\ 1.00)
\end{aligned}
$$

Individual effects

Differences between individuals were defined by specifying the primary pharmacokinetic parameters in one variance-covariance matrix, and $\mathrm{v_B}$ and σ on their own.

$$\begin{bmatrix} \tau_{K_1} \\ \tau_{V_{\text{ND}}} \\ \tau_{BP_{\text{ND}}} \\ \tau_{k_4} \end{bmatrix} \sim \text{MVNormal}\left(\begin{bmatrix} 0 \\ 0 \\ 0 \\ 0 \end{bmatrix}, \mathbf{\Sigma}_{\text{Subject}} \right)$$

$$\Sigma_{\text{Subject}} = \begin{bmatrix} \sigma_{K_1} & 0 & 0 \\ 0 & \ddots & 0 \\ 0 & 0 & \sigma_{k_4} \end{bmatrix} \mathbf{R}_{\text{Subject}} \begin{bmatrix} \sigma_{K_1} & 0 & 0 \\ 0 & \ddots & 0 \\ 0 & 0 & \sigma_{k_4} \end{bmatrix}$$

$$\tau_{\text{v}_\text{B}} \sim \text{Normal}(0, \sigma^2_{\text{Subject},\text{v}_\text{B}})$$

$$\tau_{\sigma} \sim \text{Normal}(0, \sigma^2_{\text{Subject},\sigma})$$

$$\begin{aligned} \sigma_{K_1} &\sim \text{Half-Normal}(0\ ,\ 0.3) \\ \sigma_{V_{\text{ND}}} &\sim \text{Half-Normal}(0\ ,\ 0.3) \\ \sigma_{BP_{\text{ND}}} &\sim \text{Half-Normal}(0\ ,\ 0.3) \\ \sigma_{k_4} &\sim \text{Half-Normal}(0\ ,\ 0.3) \\ \mathbf{R}_{\text{Subject}} &\sim \text{LKJ}(1) \end{aligned}$$

$$\sigma_{\text{v}_\text{B}} \sim \text{Half-Normal}(0\ ,\ 0.5)$$

$$\sigma_{\sigma} \sim \text{Half-Normal}(0\ ,\ 0.3)$$

Regional effects

For logBP_{ND} and logK_1, regional differences were defined as unpooled effects using a dummy (indicator) variable defined with reference to the dorsolateral prefrontal cortex as covariates. For simplicity, all regional differences were defined as zero-centred regularising priors with the same SD.

$$\begin{aligned} \beta_{K_1[k-1]} &\sim \text{Normal}(0\ ,\ 0.3) \\ \beta_{BP_{\text{ND}}[k-1]} &\sim \text{Normal}(0\ ,\ 0.3) \end{aligned}$$

For the remaining parameters, regional differences were defined as pooled variables, arising from a common distribution

$$
\begin{bmatrix} \upsilon_{V_{\mathrm{ND}}} \\ \upsilon_{k_4} \end{bmatrix} \sim \mathrm{MVNormal}\left(\begin{bmatrix} 0 \\ 0 \end{bmatrix}, \mathbf{\Sigma}_{\mathrm{Region}} \right)
$$

$$
\Sigma_{\mathrm{Region}} = \begin{bmatrix} \sigma_{V_{\mathrm{ND}}} & 0 \\ 0 & \sigma_{k_4} \end{bmatrix} \mathbf{R}_{\mathrm{Region}} \begin{bmatrix} \sigma_{V_{\mathrm{ND}}} & 0 \\ 0 & \sigma_{k_4} \end{bmatrix}
$$

$$
\upsilon_{\mathrm{v_B}} \sim \mathrm{Normal}(0, \sigma^2_{\mathrm{Region,v_B}})
$$

$$
\upsilon_{\sigma} \sim \mathrm{Normal}(0, \sigma^2_{\mathrm{Region},\sigma})
$$

$$
\begin{aligned}
\sigma_{V_{\mathrm{ND}}} &\sim \text{Half-Normal}(0\ ,\ 0.1) \\
\sigma_{V_{\mathrm{ND}}} &\sim \text{Half-Normal}(0\ ,\ 0.1) \\
\mathbf{R}_{\mathrm{Region}} &\sim \mathrm{LKJ}(2)
\end{aligned}
$$

$$
\sigma_{\mathrm{Region,v_B}} \sim \text{Half-Normal}(0\ ,\ 0.1)
$$

$$
\sigma_{\mathrm{Region},\sigma} \sim \text{Half-Normal}(0\ ,\ 0.5)
$$

TAC effects

Residual TAC effects were defined for the major four pharmacokinetic parameters, but were not included for $\mathrm{v_B}$ or σ as these were not considered to be of central importance.

$$
\begin{bmatrix} \phi_{K_1} \\ \phi_{V_{\mathrm{ND}}} \\ \phi_{BP_{\mathrm{ND}}} \\ \phi_{k_4} \end{bmatrix} \sim \mathrm{MVNormal}\left(\begin{bmatrix} 0 \\ 0 \\ 0 \\ 0 \end{bmatrix}, \mathbf{\Sigma}_{\mathrm{TAC}} \right)
$$

$$
\Sigma_{\mathrm{TAC}} = \begin{bmatrix} \sigma_{K_1} & 0 & 0 \\ 0 & \ddots & 0 \\ 0 & 0 & \sigma_{k_4} \end{bmatrix} \mathbf{R}_{\mathrm{TAC}} \begin{bmatrix} \sigma_{K_1} & 0 & 0 \\ 0 & \ddots & 0 \\ 0 & 0 & \sigma_{k_4} \end{bmatrix}
$$

$$
\begin{aligned}
\sigma_{K_1} &\sim \text{Half-Normal}(0\ ,\ 0.025) \\
\sigma_{V_{\mathrm{ND}}} &\sim \text{Half-Normal}(0\ ,\ 0.025) \\
\sigma_{BP_{\mathrm{ND}}} &\sim \text{Half-Normal}(0\ ,\ 0.025) \\
\sigma_{k_4} &\sim \text{Half-Normal}(0\ ,\ 0.025) \\
\mathbf{R}_{\mathrm{TAC}} &\sim \mathrm{LKJ}(2)
\end{aligned}
$$

Smooth sigma function

The smooth basis function makes use of a penalised regression spline. The $\sigma_{\text{spline-coefficients}}$ term refers to the standard deviation of the spline coefficients, which penalises the wiggliness of the spline. The $\alpha_{\text{spline-coefficients}}$ term refers to the fixed effects term, which describes the magnitude of the influence of the smooth term around the estimated mean value.

$$\sigma_{\text{spline-coefficients}} \sim \text{Half-Student-t}(\ 3,\ 0,\ 2.5)$$
$$\alpha_{\text{spline-coefficients}} \sim \text{Half-Student-t}(\ 3,\ 0,\ 4.0)$$

Covariates

For the simulations, there was only one additional covariate for group effects on BP_{ND}. For this, we used a zero-centred regularising prior. This prior conservatively assumes that no difference is most likely, and assigns 95% of its probability between differences of -48% and 48% differences between groups.

$$\beta_{\text{Group},BP_{\text{ND}}} \sim \text{Normal}(\ 0\ ,\ 0.2\ )$$

Analysis data

For the analysis, we included more covariates due to variation in the data.

Covariates

We included effects of age and sex covariates for the estimation of K_1, V_{ND} and BP_{ND}. For age, we divided ages by 10, so that it was not defined by years, but by decades. This makes it easier to specify priors, and is expected to improve efficiency of estimation by estimating parameters in a similar order of magnitude.

$$\beta_{\text{Age},K_1} \sim \text{Normal}(\ 0\ ,\ 0.1\ )$$
$$\beta_{\text{Age},BP_{\text{ND}}} \sim \text{Normal}(\ 0\ ,\ 0.1\ )$$
$$\beta_{\text{Age},V_{\text{ND}}} \sim \text{Normal}(\ 0\ ,\ 0.1\ )$$

$$\beta_{\text{Sex},K_1} \sim \text{Normal}(\ 0\ ,\ 0.05\ )$$
$$\beta_{\text{Sex},BP_{\text{ND}}} \sim \text{Normal}(\ 0\ ,\ 0.05\ )$$
$$\beta_{\text{Sex},V_{\text{ND}}} \sim \text{Normal}(\ 0\ ,\ 0.05\ )$$

Covariates for the measurement error included the centred natural logarithm of the average region size, as well as the centred natural logarithm of the injected radioactivity.

$$\beta_{\text{RegionSize},\sigma} \sim \text{Normal}(\ 0\ ,\ 0.3\ )$$
$$\beta_{\text{InjectedRadioactivity},\sigma} \sim \text{Normal}(\ 0\ ,\ 0.5\ )$$

Because so much of the variation in the measurement error between regions would be explained by region size, we reduced the variance of the prior for the partial pooling of measurement error across regions.

$$\sigma_{\text{Region},\sigma} \sim \text{Half-Normal}(0\ ,\ 0.1)$$

Supplementary Materials S2 : Simulated parameters

Global Intercepts

Parameter	Mean
$\log(K_1)$	-2.33
$\log(BP_{\mathrm{ND}})$	1.75
$\log(V_{\mathrm{ND}})$	-0.62
$\log(k_4)$	-3.84
$\log(v_{\mathrm{B}})$	-3.63
$\log(\sigma)$	-4.12

Individual effects

Standard deviations

Parameter	SD
$\log(K_1)$	0.27
$\log(BP_{\mathrm{ND}})$	0.33
$\log(V_{\mathrm{ND}})$	0.39
$\log(k_4)$	0.12
$\log(v_{\mathrm{B}})$	0.67
$\log(\sigma)$	0.42

Correlation Matrix

Parameter	$\log(K_1)$	$\log(BP_{\mathrm{ND}})$	$\log(V_{\mathrm{ND}})$	$\log(k_4)$
$\log(K_1)$	1.00	0.16	0.60	-0.25
$\log(BP_{\mathrm{ND}})$	0.16	1.00	-0.56	-0.32
$\log(V_{\mathrm{ND}})$	0.60	-0.56	1.00	0.02
$\log(k_4)$	-0.25	-0.32	0.02	1.00

Regional effects

Mean values

Here are the regional means. For simplicity of interpretation, I have added the global mean value to all of the deviations from the mean so that they are displayed as the values themselves.

Region	$\log(K_1)$	$\log(BP_{\mathrm{ND}})$	$\log(V_{\mathrm{ND}})$	$\log(k_4)$	$\log(v_{\mathrm{B}})$	$\log(\sigma)$
ACC	-2.37	1.90	-0.67	-3.75	-3.48	-4.23
Amygdala	-2.66	1.85	-0.62	-3.92	-3.63	-4.00
PCC	-2.35	1.75	-0.72	-3.72	-3.50	-4.13
DLPFC	-2.33	1.75	-0.72	-3.67	-3.70	-4.42
Hippocampus	-2.55	1.93	-0.33	-4.06	-3.70	-4.12
Insula	-2.37	2.11	-0.61	-3.83	-3.46	-4.18

Region	$\log(K_1)$	$\log(BP_{\mathrm{ND}})$	$\log(V_{\mathrm{ND}})$	$\log(k_4)$	$\log(v_{\mathrm{B}})$	$\log(\sigma)$
MPFC	-2.34	1.78	-0.68	-3.70	-3.58	-4.35
Parahippocampus	-2.60	1.76	-0.26	-3.75	-3.61	-4.22
DRN	-2.63	1.72	-0.93	-4.14	-4.03	-3.43

TAC effects

Standard deviations

Parameter	SD
$\log(K_1)$	0.053
$\log(BP_{\mathrm{ND}})$	0.037
$\log(V_{\mathrm{ND}})$	0.054
$\log(k_4)$	0.015

Correlation Matrix

Parameter	$\log(K_1)$	$\log(BP_{\mathrm{ND}})$	$\log(V_{\mathrm{ND}})$	$\log(k_4)$
$\log(K_1)$	1.00	-0.21	0.66	-0.35
$\log(BP_{\mathrm{ND}})$	-0.21	1.00	0.40	0.71
$\log(V_{\mathrm{ND}})$	0.66	0.40	1.00	0.28
$\log(k_4)$	-0.35	0.71	0.28	1.00

Smooth sigma function

The smooth sigma function across time is depicted below.

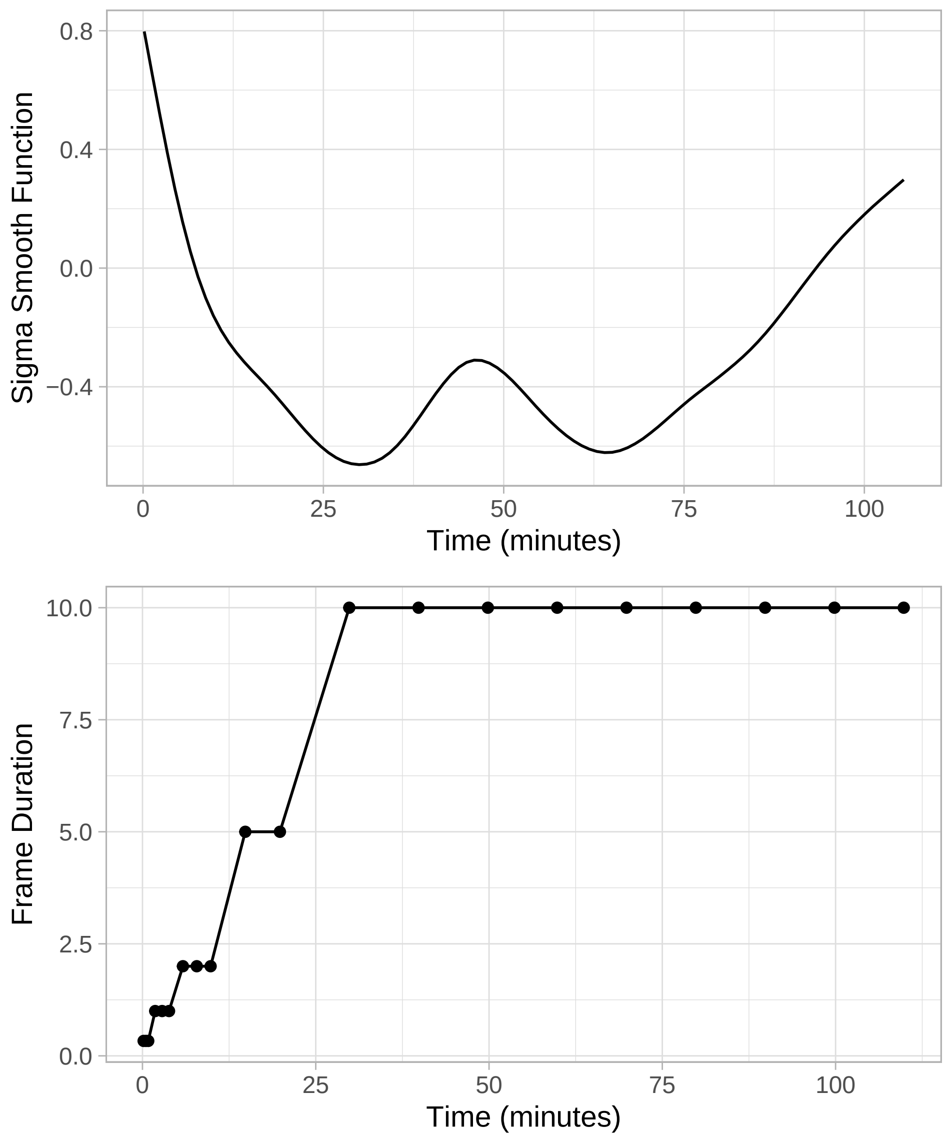

Sigma Smooth Function
0.8
0.4
0.0
−0.4
0
25
50
75
100
Time (minutes)
Frame Duration
10.0
7.5
5.0
2.5
0.0
0
25
50
75
100
Time (minutes)

Supplementary Materials S3: Comparisons between power estimation approaches

In Figures 1 and 2, we compare the outcomes estimated using the following datasets:

1. **Large Empirical:** 1000 simulated studies, with power and false positive rate calculated empirically. These datasets were only used with NLS estimation.
2. **Small Empirical:** 50 simulated studies, with power and false positive rate calculated empirically.
3. **Small Modelled:** 50 simulated studies, with power and false positive rate calculated using logspline functions applied to the confidence or credible intervals.

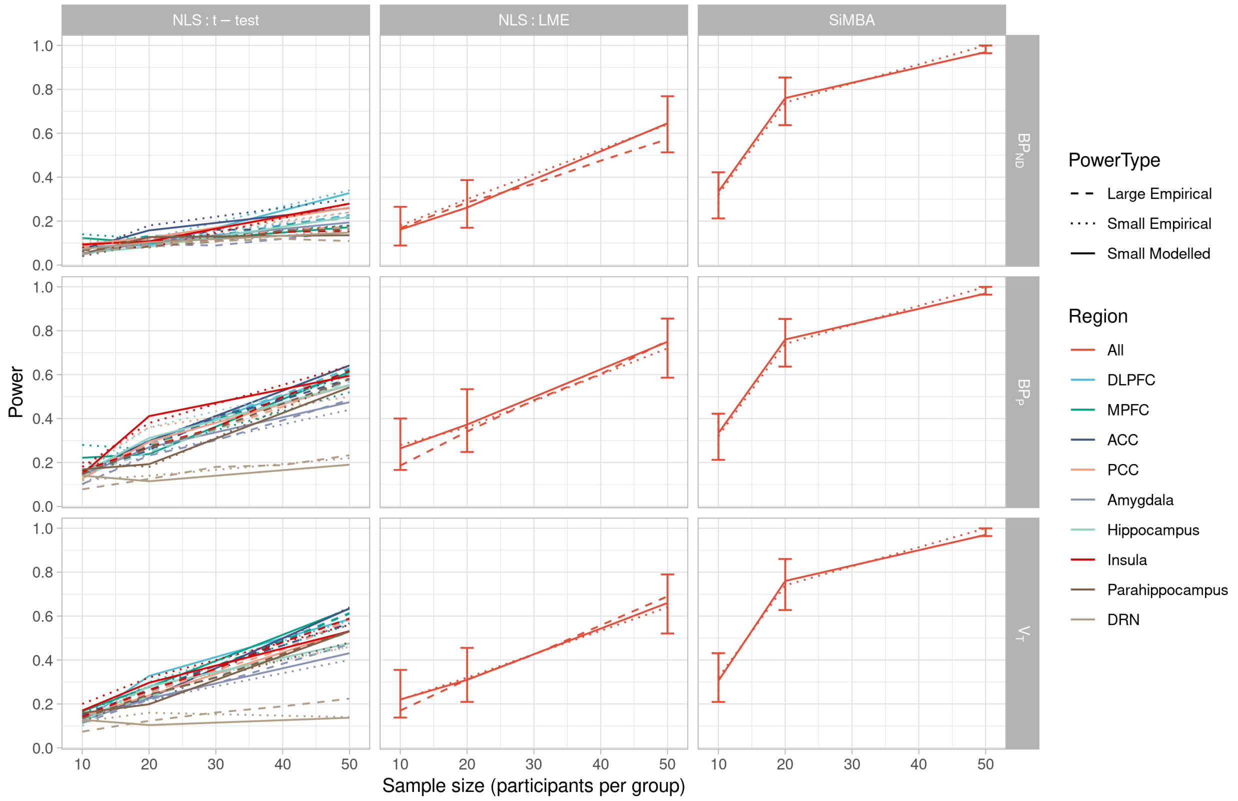

Figure 1: Power estimated using the same and different datasets between methods, with power calculated with different methods. All methods produce similar results. Error bars are not included for NLS: t-test figures for clarity.

In Figure 3 are the fits of the logspline function to the SiMBA 95% credible intervals for the calculation of the small empirical power and false positive rates.

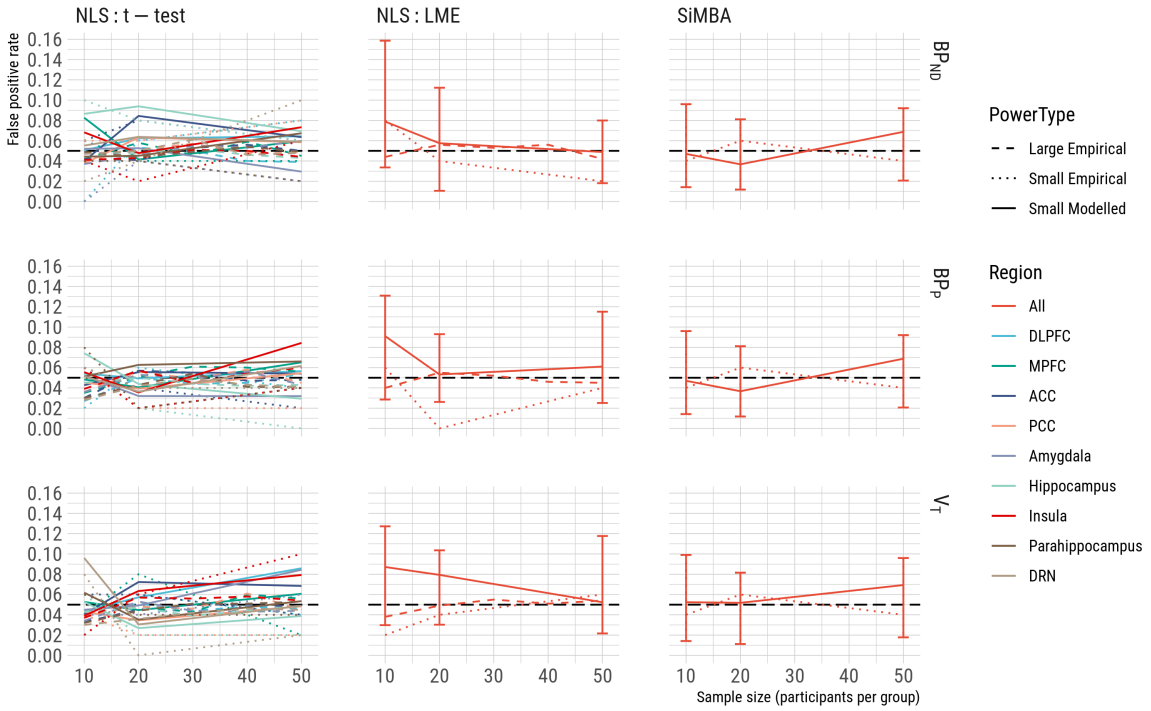

Figure 2: False positive rate estimated using the same and different datasets between methods, with the rate calculated with different methods. All methods produce similar results. Error bars are not included for NLS: t-test figures for clarity.

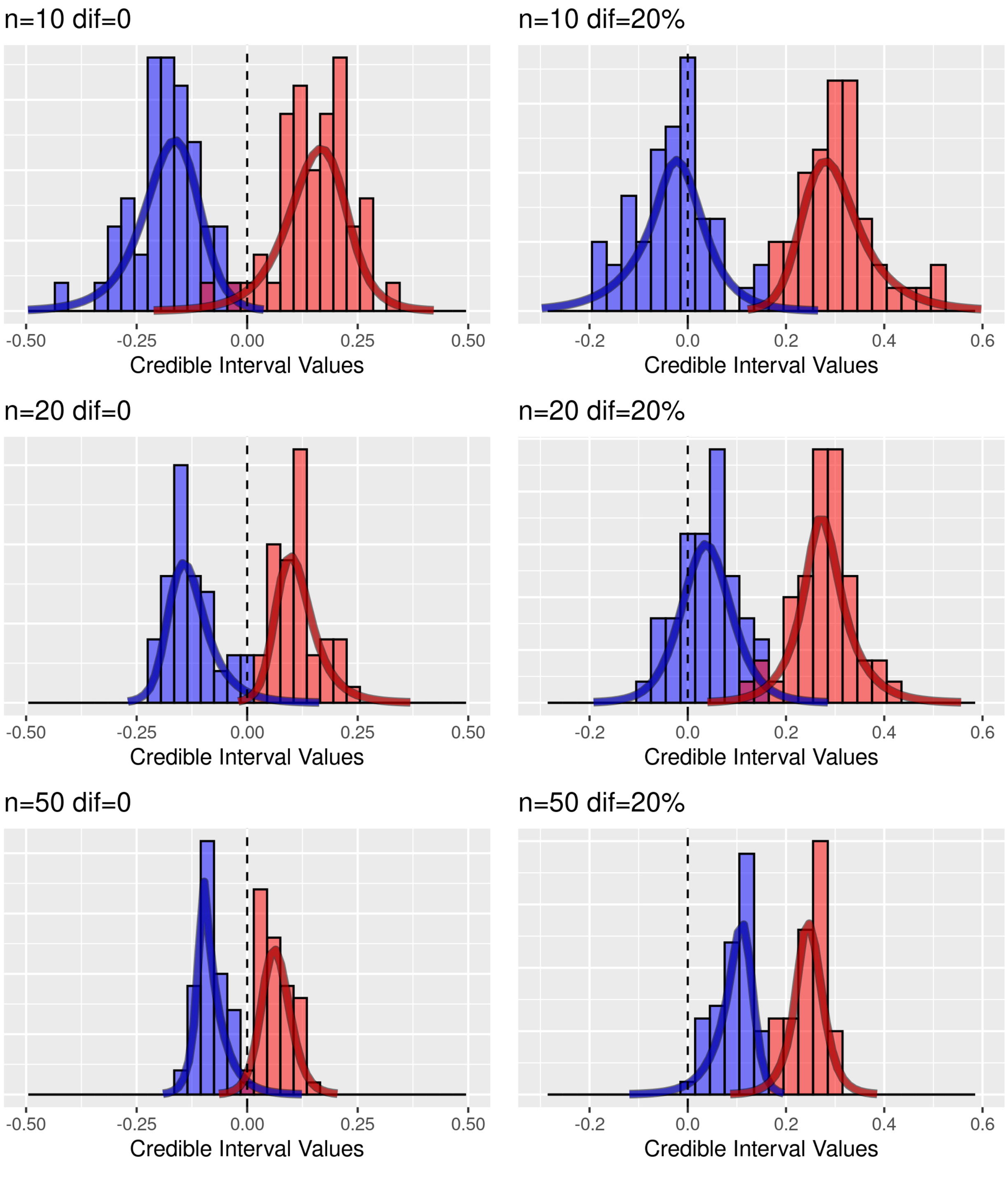

Figure 3: Fitted logspline density plots, from which power and false positive rates were calculated. Blue histograms represent the lower bound of the credible interval (i.e. the 2.5% quantile of the samples) and the red histograms represent the upper bound of the credible intervals (i.e. the 97.5% quantile of the samples).

Supplementary Materials S4: Group difference properties in the same data

Calculated in the same set of 50 datasets for each condition, we examined the mean of the 50 estimated differences, the standard deviation of the means across these datasets, the mean standard error of these estimated means across the datasets, and the (modelled) power and false positive rates. In the Figures 4 and 5, we show plots in the style of the power and false positive plots of the standard deviation and standard error of these methods in the same datasets.

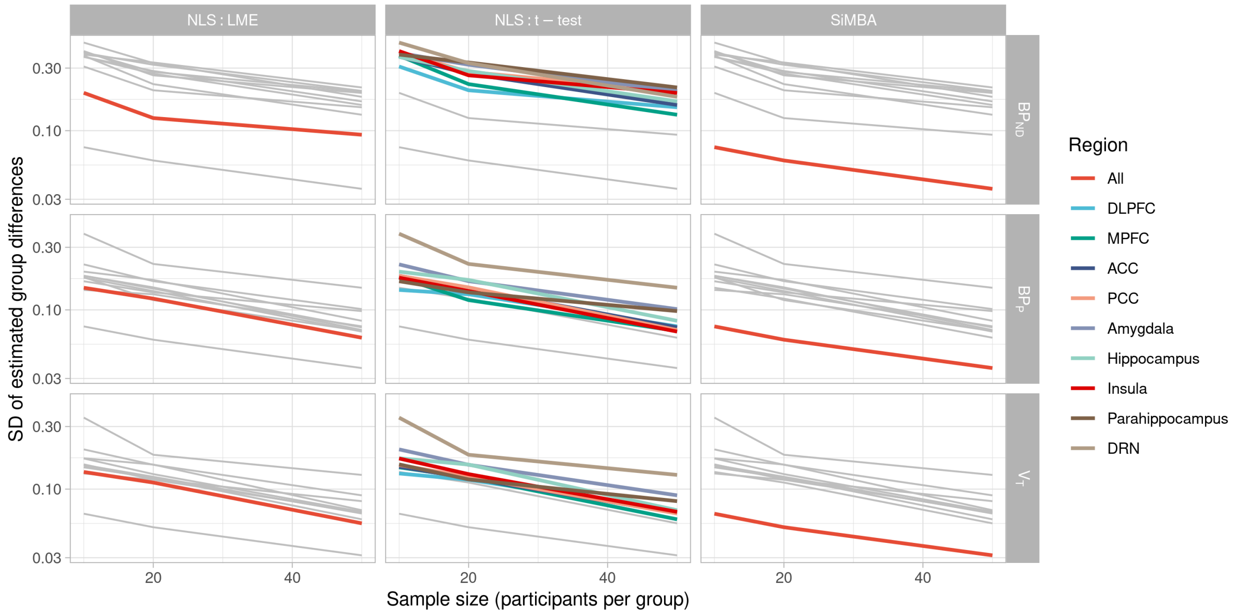

Figure 4: Standard deviation of estimated group differences across simulated datasets.

The following table includes all the data together.

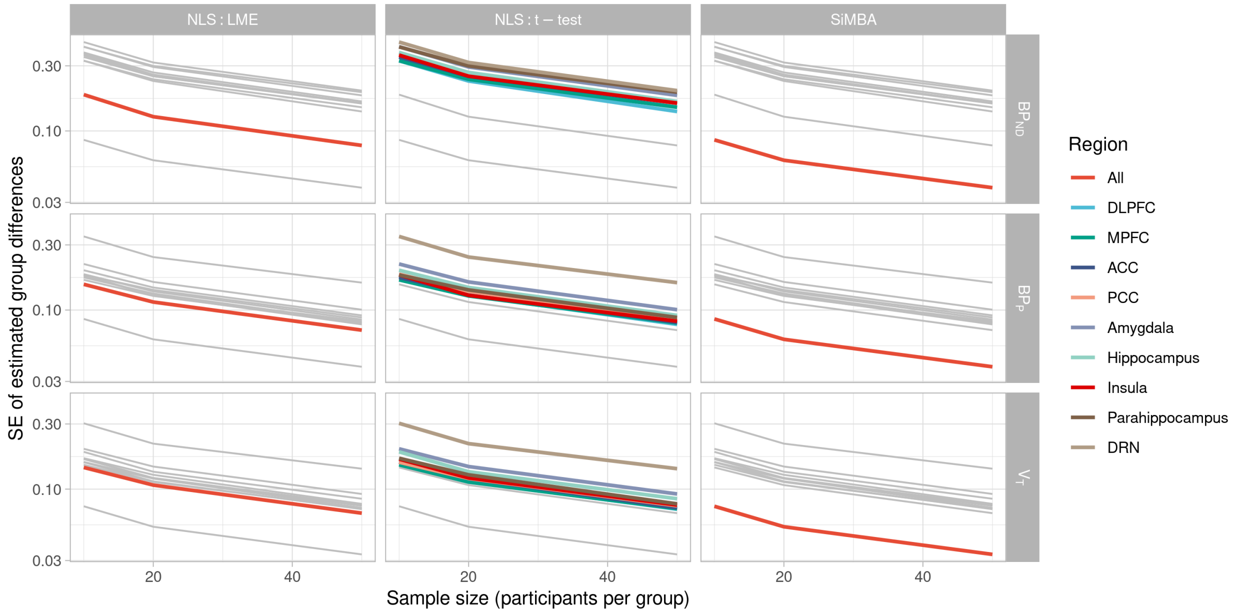

Figure 5: Mean standard error of estimated group differences across simulated datasets.

		NLS: LME				SiMBA			
n	Outcome	Mean	SD	SE	Power	Mean	SD	SE	Power
**False Positives**									
10	BPND	-0.006	0.196	0.180	0.079	-0.014	0.076	0.085	0.047
10	BPP	0.004	0.183	0.163	0.091	-0.014	0.076	0.085	0.047
10	VT	0.002	0.173	0.153	0.087	-0.012	0.065	0.072	0.052
20	BPND	0.002	0.114	0.126	0.058	-0.005	0.052	0.061	0.037
20	BPP	0.011	0.107	0.113	0.053	-0.005	0.052	0.061	0.037
20	VT	0.012	0.102	0.105	0.079	-0.004	0.045	0.052	0.052
50	BPND	0.008	0.074	0.079	0.049	-0.008	0.035	0.038	0.069
50	BPP	0.008	0.072	0.073	0.061	-0.008	0.035	0.038	0.069
50	VT	0.006	0.068	0.068	0.052	-0.007	0.030	0.032	0.069
**Power**									
10	BPND	0.156	0.192	0.182	0.166	0.142	0.075	0.086	0.334
10	BPP	0.215	0.145	0.153	0.275	0.142	0.075	0.086	0.334
10	VT	0.191	0.133	0.144	0.268	0.124	0.065	0.075	0.305
20	BPND	0.170	0.120	0.129	0.229	0.161	0.059	0.061	0.760
20	BPP	0.190	0.121	0.115	0.352	0.161	0.059	0.061	0.760
20	VT	0.167	0.113	0.107	0.317	0.139	0.051	0.053	0.760
50	BPND	0.181	0.094	0.079	0.634	0.170	0.036	0.038	0.970
50	BPP	0.183	0.060	0.071	0.764	0.170	0.036	0.038	0.970
50	VT	0.157	0.055	0.067	0.676	0.147	0.031	0.033	0.970

Supplementary Materials S5 : SD of estimated mean differences by method

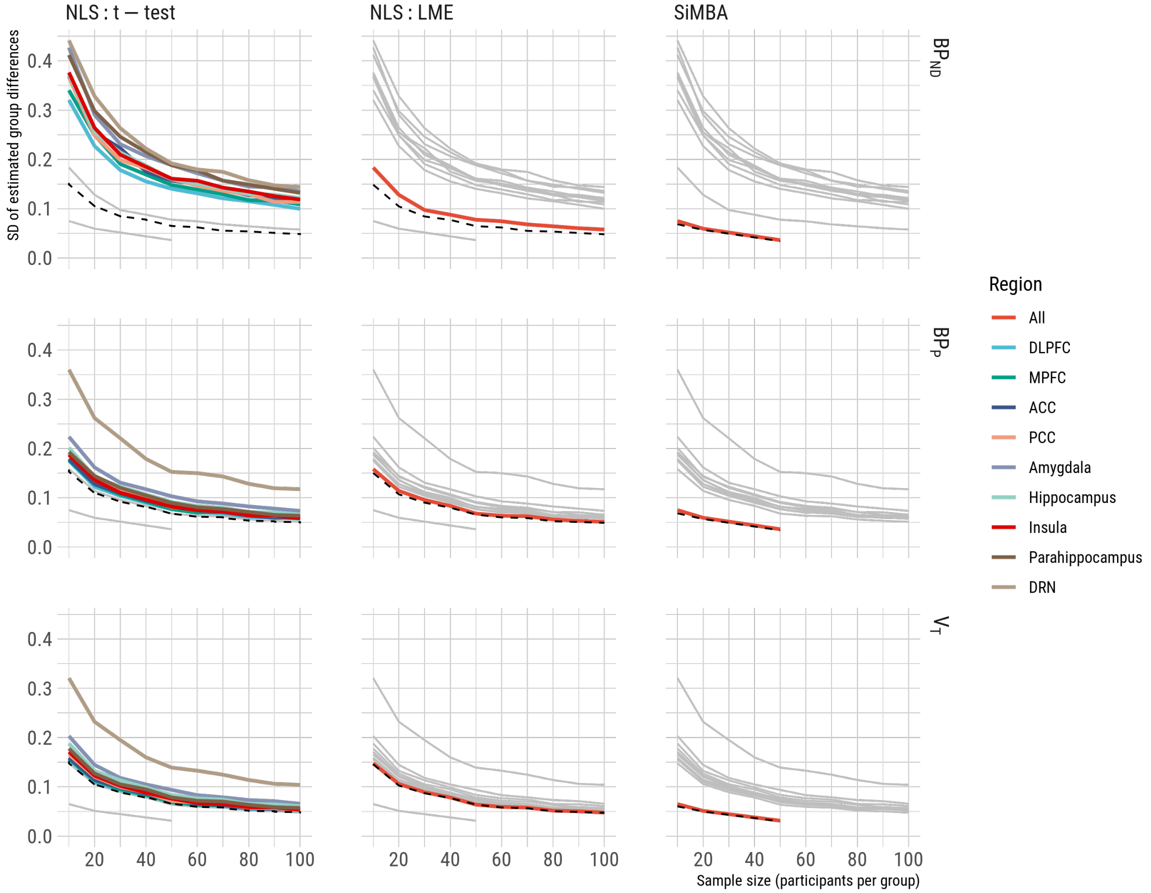

Figure 6: The standard deviation of estimated mean group differences for different sample sizes as a measure of consistency. With larger samples, the standard deviation of the estimates is decreased, indicating greater consistency of estimates. Linear mixed effects (LME) modelling of binding outcomes show less variation than t-tests, and the Bayesian hierarchical pharmacokinetic modelling of TACs has greatly reduced variation compared to both LME modelling and t-tests.

Supplementary Materials S6: Sensitivity to measurement error

In Figure 7, we show the power (estimated using logsplines and bootstrapped 95% confidence intervals), the mean posterior estimates of α_σ, the standard deviation (SD) of the mean posterior estimates of the group differences (with 95% confidence intervals estimated using a case resampling bootstrap procedure), and the mean standard error (SE) of the differences from within each simulated study (with 95% quantiles). All values are calculated for BP_{ND} and BP_P (as these two are the same due to not estimating different V_{ND} values between groups).

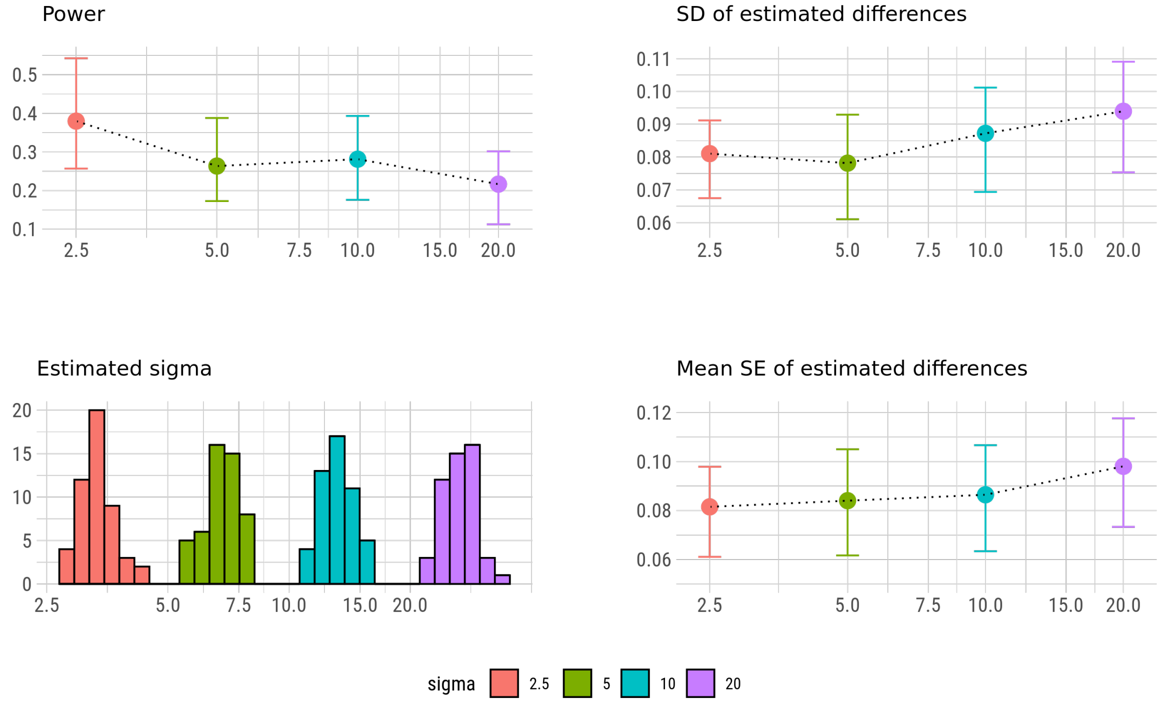

Figure 7: The SiMBA model is sensitive to measurement error, but even with the standard deviation of the measurement error equal to 20% of the mean TAC value, the power with n=10 is still over 20%.

Supplementary Materials S7: Application to data with no parameter intercorrelations

Data were simulated with the originally estimated parameter intercorrelations, as well as following sampling from univariate distributions, thereby removing the correlations between parameters. We examined the distribution of the estimated parameter intercorrelations in the original, and in the uncorrelated data. Comparisons between correlated and uncorrelated data are shown in figures 8 and 9.

Also displayed are the estimated correlations in the original data for different sample sizes in figures 10 and 11.

In all cases, the measurement error of the simulated data is 10%, which is more than twice that of the original dataset.

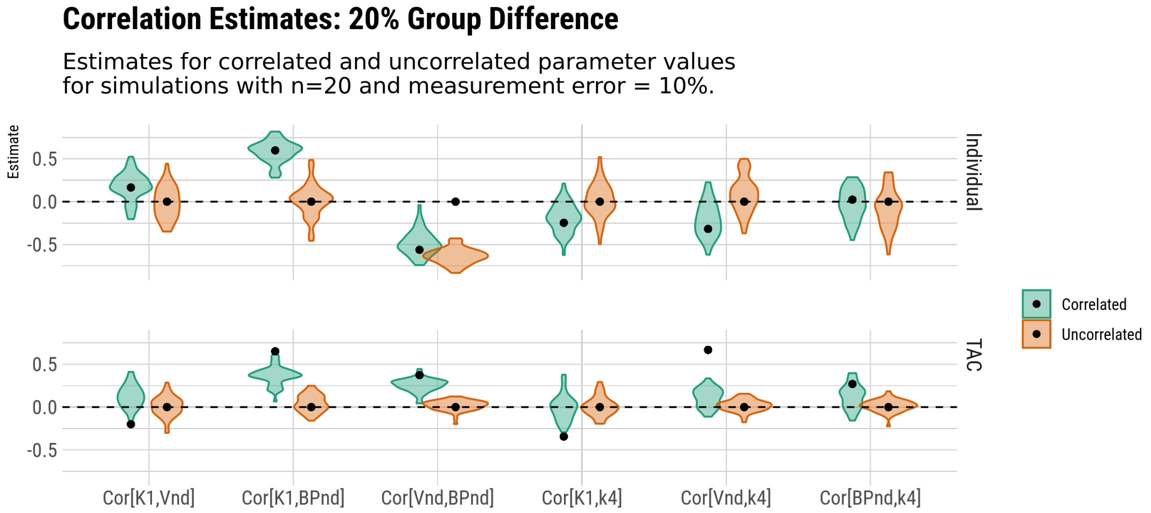

Figure 8: Estimated correlations comparing correlated and uncorrelated data in the presence of no group difference. True values are represented using black circles.

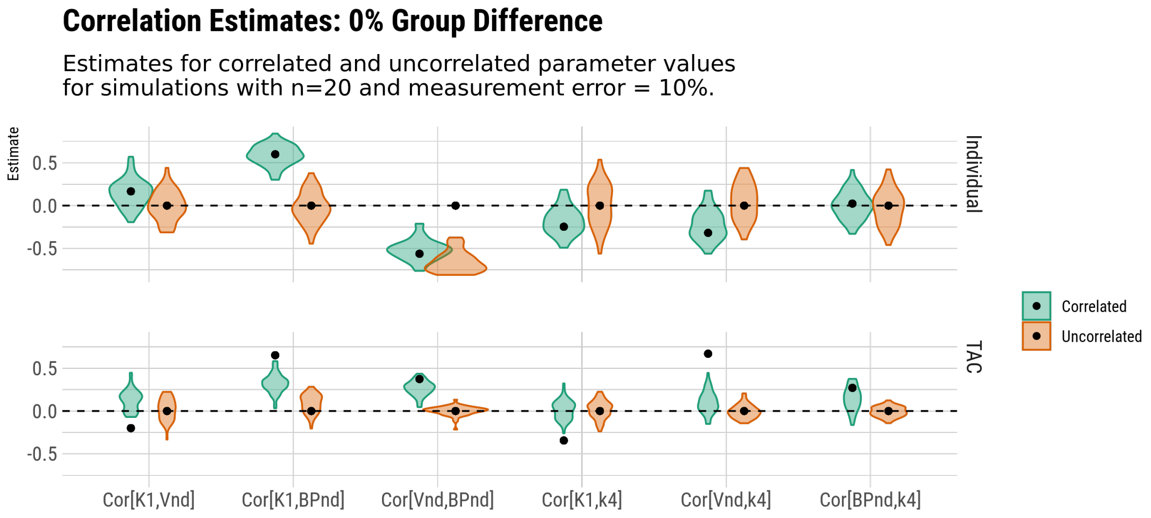

Figure 9: Estimated correlations comparing correlated and uncorrelated data in the presence of a group difference. True values are represented using black circles.

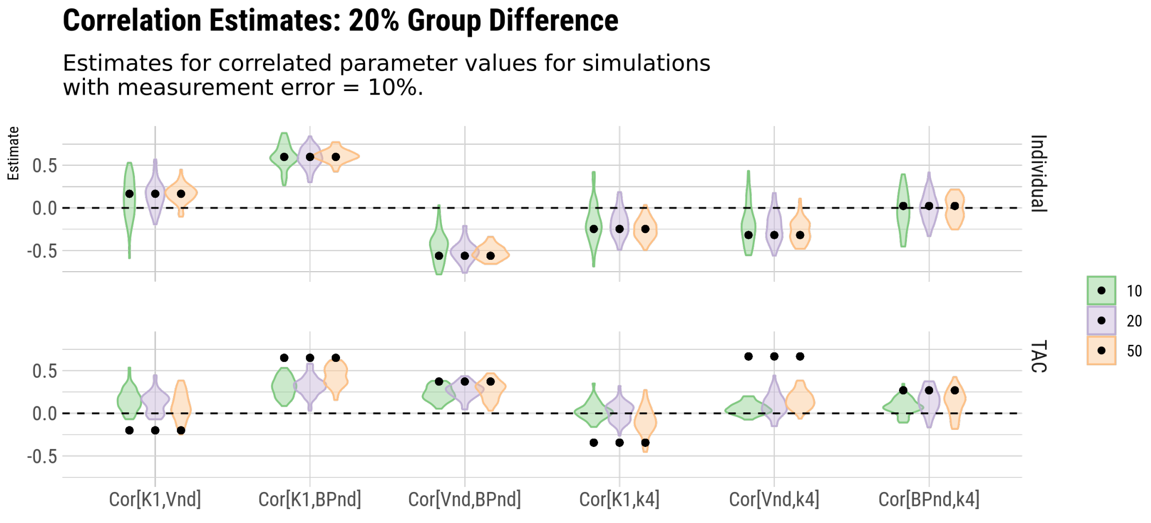

Figure 10: Estimated correlations for correlated data with different sample sizes in the presence of a group difference.

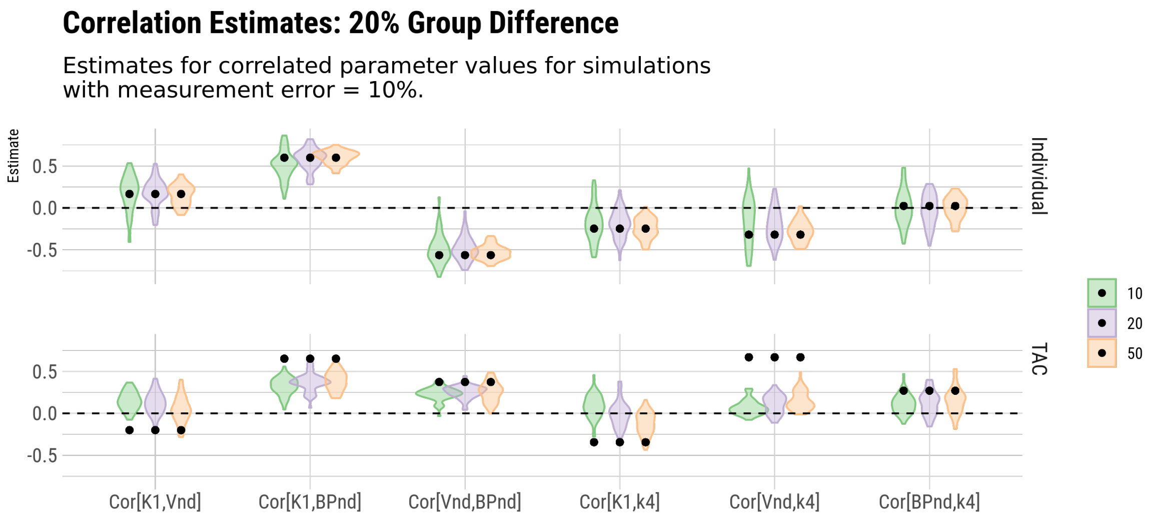

Figure 11: Estimated correlations for correlated data with different sample sizes in the presence of a group difference.
